# Metabolic plasticity in blast crisis-chronic myeloid leukaemia cells under hypoxia reduces the cytotoxic potency of drugs targeting mitochondria

**DOI:** 10.1007/s12672-022-00524-y

**Published:** 2022-07-08

**Authors:** Luciana S. Salaverry, Tomás Lombardo, María C. Cabral-Lorenzo, Martin L. Gil-Folgar, Estela B. Rey-Roldán, Laura I. Kornblihtt, Guillermo A. Blanco

**Affiliations:** 1 Department of Immunology IDEHU-CONICET, Faculty of Pharmacy and Biochemistry, University of Buenos Aires (UBA), Buenos Aires, Argentina; 2 Laboratory of Immunotoxicology (LaITo), IDEHU-CONICET, Clinics Hospital, Jose de San Martin, University of Buenos Aires (UBA), Junin 956 4to piso, Capital Federal (1113), Buenos Aires, Argentina; 3 Department of Pathology, Clinics Hospital, Jose de San Martin, University of Buenos Aires (UBA), Buenos Aires, Argentina; 4 Department of Hematology, Clinics Hospital, Jose de San Martin, University of Buenos Aires (UBA), Buenos Aires, Argentina

**Keywords:** Metabolic reprogramming, Arsenic Trioxide, Dichloroacetate, Gene expression profiling, Valproic acid, Glucose uptake

## Abstract

Metabolic reprogramming (MR) influences progression of chronic myeloid leukaemia (CML) to blast crisis (BC), but metabolic programs may change transiently in a second dimension (metabolic plasticity, MP), driven by environments as hypoxia, affecting cytotoxic potency (CPot) of drugs targeting mitochondria or mitochondria-related cell stress responses (MRCSR) such as mitophagy and mitochondrial biogenesis. We assessed mitochondrial membrane potential (MMP), mitochondrial mass (MM), apoptosis, glucose uptake (GU), and CPot of arsenic trioxide (ATO), CCCP, valproic acid (VPA), vincristine (VCR), Mdivi1, and dichloroacetic acid (DCA) in CML BC cells K562 (BC-K562) under hypoxia through flow cytometry, and gene expression from GEO database. About 60% of untreated cells were killed after 72 h under hypoxia, but paradoxically, all drugs but ATO rescued cells and increased survival rates to almost 90%. Blocking mitophagy either with VCR or Mdivi1, or increasing mitochondrial biogenesis with VPA enhanced cell-survival with increased MM. DCA increased MM and rescued cells in spite of its role in activating pyruvate dehydrogenase and Krebs cycle. Cells rescued by DCA, VPA and CCCP showed decreased GU. ATO showed equal CPot in hypoxia and normoxia. MP was evidenced by differential expression of genes (DEG) under hypoxia related to Krebs cycle, lipid synthesis, cholesterol homeostasis, mitophagy, and mitochondrial biogenesis (GSE144527). A 25-gene MP-signature of BC-K562 cells under hypoxia identified BC cases among 113 transcriptomes from CML patients (GSE4170). We concluded that hypoxic environment drove a MP change evidenced by DEG that was reflected in a paradoxical pro-survival, instead of cytotoxic, effect of drugs targeting mitochondria and MRCSR.

**Supplementary Information:**

The online version contains supplementary material available at 10.1007/s12672-022-00524-y.

## Introduction

Chronic myeloid leukaemia (CML) is caused by a chromosomal translocation that results in the expression of the fusion protein bcrabl1 with kinase activity [1]. The course of CML was radically modified with the introduction of tyrosine kinase inhibitors (TKIs) such as imatinib. However, imatinib does not cure the disease and interruption of treatment usually leads to relapse. In some cases, CML evolves from chronic phase (CP) into more advanced forms, such as accelerated phase (AP) and blast crisis (BC) [2], with resistance to TKIs due to mutations in the bcrabl1 protein. However, in many cases this type of mutation is not detected and the cause of progression remains elusive.

Metabolic reprogramming (MR) is considered a neoplastic hallmark, and involves the expression of genes associated with aberrant metabolic programs compared to the cells of origin [3]. MR is a phenotypic trait or dimension that evolves along disease progression. For example, metabolic pathway genes may show different profiles of expression in BC cells, AP cells, and CP cells [4]. However, alternative metabolic programs are not permanently active but may change in a second phenotypic dimension according to the microenvironment. This rather transient changes are often referred to as metabolic plasticity (MP), and allow neoplastic cells to tolerate temporary harsh conditions such as lack of oxygen or nutrients (glucose, or amino acids such as glutamine) [5]. For example, mitochondrial and metabolic pathway genes may show different profile of expression in BC cells under hypoxia compared to the same cells under normoxia. Therefore, MR and MP are independent phenotypic dimensions, with the latter being driven by transient changes in the microenvironment. Increasing MP is supported by anabolic and catabolic pathways that largely interconnect at the mitochondrial level. The most direct example of MP is the shift to glycolysis as the main source of ATP under hypoxia, to compensate reduced oxidative phosphorylation (OXPHOS) at the mitochondria. However, mitochondria can assist biosynthetic pathways, where the Krebs cycle functions to provide metabolites that are exported to the cytoplasm for the synthesis of nucleotides, fatty acids, cholesterol, amino acids, and anti-oxidant molecules [3, 6].

Mitochondria initiate apoptosis and are the target of most cytotoxic drugs [7]. Being at the intersection of metabolic and apoptosis control, mitochondria may influence the threshold of cytotoxic drugs, depending on the microenvironment and the metabolic program adopted by cells [8]. Cytotoxic drugs must alter mitochondrial function sufficiently enough to reach the threshold of apoptosis initiation and kill cells. For this reason, metabolic reprogramming, and more specifically the metabolic pathways regulated by mitochondria, can significantly modify the effect of cytotoxic drugs [9]. In other words, MR along disease progression, and MP between different environments, can not only be observed at transcriptomic, proteomic or metabolic dimensions, but can also be exposed by altered drug potency in harsh environments such as hypoxia or lack of nutrients.

The physiological response to hypoxia in normal cells is mediated by hypoxia-inducing-factor (HIF). HIF-targeted genes are known to increase glycolysis as a source of ATP, and reduce mitochondrial mass (MM) through increased mitophagy [10]. The classical view considers that mitochondria cannot produce ATP under hypoxia, and therefore their rapid elimination through mitophagy is necessary to prevent their failure, since a failing OXPHOS may lead to mitochondrial apoptosis [11]. It is often assumed that mitochondrial failure due to lack of O_2_ could increase sensitivity to drugs that activate mitochondrial apoptosis. This has established a general principle for blocking the adaptation to hypoxia of neoplasms, which consists of actions such as inhibiting mitophagy, inducing an increase in MM by targeting mitochondrial biogenesis, or forcing the resumption of OXPHOS with metabolic drugs, therefore leading to massive mitochondrial failure and onset of apoptosis [12, 13]. However, the metabolic change induced by hypoxia in advanced leukaemia could have an influence on mitochondrial apoptosis. One way of exploring the impact of microenvironment-induced MP is to explore the cytotoxic potency of drugs under hypoxia. Hypoxia below 1% induces a metabolic change, since mitochondria cannot produce enough ATP and must resort to glycolysis. CCCP is a compound that blocks OXPHOS by uncoupling electron transport chain (ETC) causing significant mitochondrial damage and inducing apoptosis [14, 15]. In the presence of CCCP it is not possible to obtain ATP via OXPHOS and the only alternative to produce ATP is glycolysis. Arsenic trioxide (ATO) is a widely studied compound that targets mitochondria and also inhibits OXPHOS, forcing glycolysis as an alternative source of ATP [16]. However, it does so not only at the level of ETC, but also by inhibiting key enzymes of the Krebs cycle such as pyruvate dehydrogenase (PDH) and 2-oxoglutarate dehydrogenase (2OGDH) [17]. Vincristine (VCR) and Mdivi1 are two drugs that inhibit mitophagy, but differ in the mode of inhibition. Mdivi1 blocks mitochondrial fission, which in turn is the first step of mitophagy, and consists of separating those mitochondria of low quality or with bioenergetic failure [18, 19]. VCR inhibits the polymerization of microtubules, preventing the transport of damaged mitochondria into lysosomes, and in particular the fusion of “mitophagosomes” with lysosomes, which is the final step of mitophagy [12, 20]. Valproic acid (VPA) is a histone deacetylase inhibitor that activates the expression of PGC1α, the main transcription factor of mitochondrial biogenesis [21], while dichloroacetic acid (DCA) is an inhibitor of pyruvate kinase-1 (PDK1) and offsets HIF-driven response to hypoxia by restoring the function of PDH, the Krebs cycle and OXPHOS [22]. All these drugs should be very toxic for adaptation to hypoxia considering the classical physiological principles of survival under low oxygen tension.

The objective of this work was to expose BC-K562 cells to conditions of hypoxia (< 1% O_2_) in order to force a metabolic change, and explore the cytotoxic effect of the six above-mentioned drugs that target mitochondria, or mitochondria-related cell stress responses (MRCSR) such as mitochondrial biogenesis, mitophagy, and cell metabolism (ATO, CCCP, Mdivi1, VCR, VPA and DCA). Our results showed several paradoxical effects of these drugs, contrasting the classical view. We interpret that these effects are caused by both the MR of BC cells, and MP driven by the hypoxic environment. We support our interpretation exploring differential expression of genes (DEG) related to mitochondria, MRCSR and metabolism in BC-K562 cells under hypoxia, as an indication of MP (MP-signature). In addition, we investigated if these genes were also differentially expressed in BC cells from CML patients compared to CP and AP patients as an indication of overall MR during CML progression (MR-signature). From the analysis of two public available gene expression data sets, we concluded that MP of BC-K562 under hypoxia promoted mitochondrial functions not related to OXPHOS but to mitochondrial anabolic pathways and MRCSR, and that these changes could be the cause of the paradoxical effect of drugs on BC-K562 under hypoxia.

## Materials and methods

### Cell model, culture conditions and reagents

The study was conducted on the cell line K562 that was originally derived from a sample of the pleural effusion of a 53-old female CML patient in BC eight days prior to her death in 1970 [23]. This cell line is positive for the BCR:ABL fusion gene (Philadelphia chromosome) and is included in a panel of the 60 cell lines selected by the National Cancer Institute of USA to identify and characterize novel anti-cancer compounds [24]. K562 cells were purchased from ATCC and grown in RPMI 1640 with 10% foetal bovine serum (FBS) at 37 °C in a 5% CO_2_ atmosphere. Cells were subcultured at 1:2 ratios every 2 to 3 days with a viability greater than 95%. For the experimental assessment of drugs, K562 cells were decanted in 24-well culture plates at 0.5 × 10^6^/ml. Serial dilutions of the corresponding single drug or combinations were added in triplicate. RPMI was added as untreated control. The plates were incubated at 37 °C with 5% CO_2_ at ambient air (21% O_2_) and were denoted as “normoxia” experimental conditions.

A second series of experiments that we denoted as “hypoxia” experimental conditions was conducted in similar culture plates but within a Modular Incubator Chamber (MIC-101) from Billups Rothenberg, Inc. (Del Mar, USA) as described previously [25]. The chamber was attached to a flow meter from the same manufacturer (SFM3001; 3–25 LPM adjustable flow rate) to regulate the gas flow. To attain hypoxia conditions after adding the drugs, the cells were immediately placed in the chamber and flushed for 15 min with a gas mixture of 5% CO_2_ and 95% N_2_. The chamber was sealed and placed at 37 °C in a conventional cell incubator, ensuring the O_2_ concentration is maintained below 1.0% up to 72 h [26, 27].

The classical view of survival under hypoxia considers that mitochondria is superfluous as source of energy under low O_2_ tension, and may pose severe risks of cell death due ETC failure. Assuming that blocking elimination of mitochondria or even increasing mitochondrial network would be severely deleterious for cell survival under hypoxia, we exposed BC-K562 cells to conditions of hypoxia in order to force a metabolic change, and explore the cytotoxic effect of six drugs that target mitochondria and MRCSR, such as mitochondrial biogenesis (VPA), mitophagy through microtubule transport inhibition (VCR) and blockage of mitochondrial fission (Mdivi1), disruption of ETC and OXPHOS without compromise of Krebs cycle (CCCP), disruption of ETC and Krebs cycle (ATO), and disruption of Krebs cycle with offset of HIF1-mediated response by increased PDH activity (DCA).

Carbonyl cyanide 3-chlorophenylhydrazone (CCCP), and 3-(2,4-Dichloro-5-methoxyphenyl)-2,3-dihydro-2-thioxo-4(1H)-quinazolinone (Mdivi-1) were from Sigma (Buenos Aires, Argentina). Stock solutions were prepared in dimethylsulfoxide (DMSO) at 80 mM and 5 mM concentration respectively. Valproic acid was from Casasco Pharmaceuticals (Argentina) and was prepared freshly as a 100 mM stock solution in RPMI-1640. Vincristine was kindly provided by Laboratorio Filaxis (Argentina). Arsenic trioxide (ATO) was a kind gift from Varifarma (Argentina). Fresh stock solutions of 1 mM ATO were prepared before every assay. DCA was from Sigma-Aldrich (Buenos Aires, Argentina). Fresh stock solutions of 1 M DCA in RPMI-1640 were prepared before every assay. RPMI-1640, penicillin, streptomycin, tetramethylrhodamine-ethyl-ester (TMRE), nonyl-acridine orange (NAO), and 2-deoxyglucose analog, 2-[N-(7-nitrobenz-2-oxa-1,3-diaxol-4-yl)amino]-2-deoxyglucose (2-NBDG) were purchased from ThermoFisher, and prepared as a 10 mM stock solution in DMSO.

### Labelling of cells with fluorescent probes

We employed a flow cytometry multiparametric method to simultaneously assess mitochondrial mass (MM), mitochondrial membrane potential (MMP), and cell death in single cells as described previously [15]. Briefly, the MMP was evaluated by the potentiometric probe TMRE [12]. Cells were incubated in 0.05 μM TMRE for 20 min at 37 °C. Damage to cell membrane was detected by labelling with 1 µM propidium iodide (PI). The MM was evaluated with nonyl-acridine orange (NAO) [28]. NAO was prepared as a 5 mM stock solution in ethanol, and diluted to 100 nM in PBS to stain cells during 15 min at 37 °C. The cells were washed once in PBS and analysed by flow cytometry. To determine glucose uptake cells were labelled with 25 μM 2-NBDG during 40 min and washed once in PBS before flow cytometry analysis.

### Flow cytometry analysis

All samples were evaluated in a Partec PAS III flow cytometer equipped with a 20 mW 488 nm argon blue laser (Partec, GmbH, Münster, Germany). A total of 20,000 cells were analysed in each sample. Fluorescence from NAO and 2-NBDG was collected through a 520/10 nm bandpath (BP) filter (FL1). Fluorescence from TMRE was collected with a 590/20 nm BP filter (FL2) and PI was collected with 670/20 nm BP filter. Flow cytometry data analysis was performed with Flomax software (Partec, Germany) and Flowing Software 2.5.1 (Turku Centre for Biotechnology, Finland). Unsupervised classification of labelled cells through flow cytometry self-organized maps (FlowSOM) algorithm [29], and visualization of cells in a five-dimensional space (FSC, SSC, FL1, FL2 and FL3) through t-distributed stochastic neighbour embedding (tSNE) plots [30], was conducted with FlowJo software 10.5.0 (BD, USA) using the standard plugins.

### Assessment of the mean cytotoxic effect of single drugs

We used a quantal model of dose–response function, where the number of dead and live cells scored by flow cytometry was used to define two ratio measures. *Fa* was the fraction of cells affected by cytotoxic drugs, and *Fu* was the fraction of live cells, with *Fu* = (1−*Fa*). By linear regression, we obtained the slope and the intercept of the equation log(*Fa*/*Fu*) = m. log (D)−m. log (Dm), where D was the dose of the drug tested. With the slope m and the median effect dose Dm we obtained the median effect equation for each individual drug as: D = Dm (Fa/(1-Fa))^1/m^. With this formula, we estimated the dose D of drug causing the death of fraction *Fa* of cells after 72 h. Therefore, we referred to the *Fa* values as the cytotoxic effect (EC) and we denoted EC50 as the dose that causes death in 50% of cells [25, 31].

### Differential gene expression

To explore changes in the expression of genes related to mitochondria and metabolism that could be induced by hypoxia in BC-K562 cells, we compared the transcriptomes of BC-K562 cells under normoxia and hypoxia, using raw data from an RNAseq dataset that became available recently in GEO database (GSE144527). This dataset includes six transcriptomes obtained from BC-K562 cells exposed to 1% O_2_ and 21% O_2_ during 72 h [32].

After obtaining a list of 25 genes related to mitochondria and metabolism from our analysis on the GSE144527, whose expression characteristically changed under hypoxia (MP-signature), we further explore if these genes were also differentially expressed in BC cells from CML patients. For this purpose we used another transcriptomic dataset from a large cohort of CML patients (GSE4170), also deposited in the GEO database. This dataset includes 113 transcriptomes from CML patients corresponding to 62 cases of CP, 17 cases of AP and 32 cases of BC [33]. We used the conventional bioinformatics tools GEOquery, limma (Linear Models for Microarray and RNA-Seq Data) and edgeR from R bioconductor to evaluate DEG and detect functional groups of genes associated with metabolism and mitochondrial function. We listed the top-ranked individual DEG using lmFit, eBayes and TopTable functions of the R limma package. Next, we listed the top-ranked Gene Ontology (GO) sets with DEG in terms of cellular component (CC), biological processes (BP), and molecular functions (MF) using GOANA functions of the limma R package, and we listed the top-ranked pathway gene sets with DEG from the Kegg pathway database using the KEGGA function of the limma R package. The entire fitted linear model (fit object) as obtained with lmFit and eBayes functions of limma package, containing all genes probed, was used as input for GOANA and KEGGA functions, using the default FDR cut-off parameter value of 0.05 [34].

### Gene set enrichment analysis and hierarchical clustering

We explored coordinated expression of genes known to be involved in particular functions through the ranks-based methods provided by the Gene Set Enrichment Analysis (GSEA) software platform, which includes a Molecular Signature Database (MSigDB) with eight major collections of gene sets. We used the Hallmark collection of gene sets that consists of 50 sets of well-defined biological states or processes with little overlap between them, to obtain an unbiased overview of cell states under hypoxia and normoxia in BC-K562 cells.

The GSEA analysis is not restricted to the list of genes with differential expression above a certain level of statistical significance, but instead performs an evaluation using all genes for which the dataset has available RNA expression values. In this way GSEA can track even several minor changes occurring simultaneously in specific functional gene lists, using a statistical method based on ranks. The ranked differential expression list was built using the default ranking metric “signal-to-noise ratio” of the GSEA software, which is defined as S2N = (x̄1−x̄2)/(s1−s2), where for each gene, x̄1 and s1 corresponded to the average and standard deviation of gene expression of three samples under hypoxia, and x̄2 and s2 corresponded to the average and standard deviation of three samples under normoxia [35]. Nominal p-values and q-false discovery rates (FDR) probability measures were used as statistical significance values of gene set enrichment, as calculated by GSEA software [35]. We restricted the analysis to 4362 genes (from the large set of 21,950 genes) whose expression was consistently detected in all 6 samples of GSE144527 (three replicates of BC-K562 cells in normoxia, and three replicates in hypoxia), considering genes with at least 10 counts from the RNAseq assessment in all 6 samples. In this way, we constrained the exploration of enrichment to genes with clear expression in BC-K562 cells under normoxia and hypoxia.

We further defined gene sets potentially associated to metabolic plasticity from GSEA and DEG analysis, and we conducted unsupervised classification through hierarchical clustering. Classification was conducted on gene expression values in transcriptomes from GSE144527 and GSE4170 using hclust and dendextend packages from R-Bioconductor, for computing the Euclidean distance and applying the complete linkage method. Additional R packages used for hierarchical clustering analysis and heatmaps production were gplots, factoextra, and dendextend [36].

### Statistics

In DEG analysis a p-value below 0.05% was considered significant. An adjusted p-value < 0.05 was computed through the Benjamini-Horchberg method to consider false positives rates (FDR) due to multiple comparisons. Bars in bar graphs in figures and supplemental materials indicate SE of the mean fluorescence intensity or fraction of cells from three replicates. In all cases, bar graphs show one experiment representative of at least three. Spearman-rank correlation analysis and contingency tables analysis were performed with GraphPad Prism 7.0 (GraphPad Software, Inc.).

## Results

### Cytotoxicity of ATO on BC-K562 cells under normoxia

**Fig. 1:**
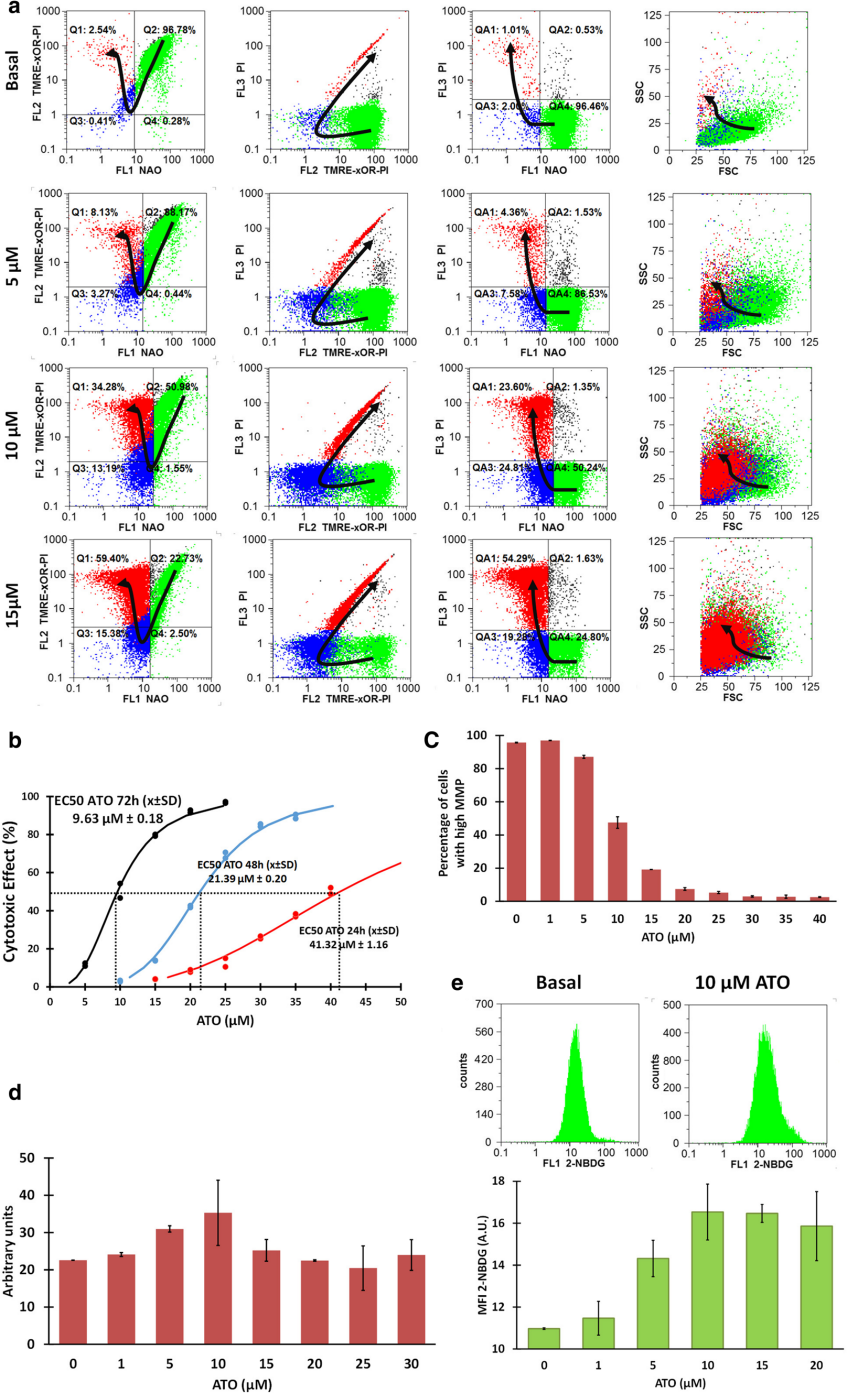
Cytotoxic effect of ATO under normoxia in BC-K562 cells. **a** Flow cytometry analysis of triple-stained ATO-treated cells. Bivariate dot plots show representative samples with increasing doses of ATO. The arrows indicate the phenotypic transition with increasing ATO doses. The green dots match viable cells with rdCL (NAO-high). The blue dots match live apoptotic cells with oxCL (NAO-low) and without plasma membrane damage (PI-). The red dots match dead cells with membrane damage (PI +) and oxCL (NAO-low). The Y axis in the first column shows both the MMP (TMRE-high) with rdCL, and the membrane damage (PI +) with oxCL (green and red clusters), since dead cells never have MMP and high MMP occurs only in live cells (xOR indicates mutual exclusion). This is illustrated in 2nd and 3rd column (MMP vs PI, and NAO vs PI respectively) where green and red dots are clearly separated. The 4th column shows the characteristic morphological changes of apoptosis according to size (FSC) and granularity (SSC). **b** Regression dose–response curve using the median effect equation, with EC50 of ATO obtained at 24, 48, and 72 h treatment. Cytotoxic effect at each dose was computed from percentages in Q1 + Q3. **c** Percentage of cells with high MMP at 72 h observed at increasing doses of ATO. **d** MM of cells at 72 h at increasing doses of ATO. **e** Two representative histograms of cells labelled with glucose uptake indicator 2-NBDG, and bar graph showing increased glucose uptake with increasing doses of ATO. MFI: mean fluorescence intensity; A.U. arbitrary units

We evaluated drug-induced changes in MM, MMP, and apoptosis on BC-K562 cells using a flow cytometry triple-staining method with NAO-TMRE and PI. NAO produces bright fluorescence when bound to reduced-cardiolipin (rdCL) at the mitochondria, but emits low fluorescence when bound to oxidized-cardiolipin (oxCL) [28]. The abrupt drop in NAO intensity with collapsed MMP (TMRE low) is an indicator of ongoing apoptosis with mitochondrial outer membrane permeabilization (MOMP), massive oxidation and passage from rdCL to oxCL and release of cytochrome c. Increasing doses of ATO at 72 h caused a gradual decline in MMP with rdCL, followed by an increase in oxCL without cell membrane damage, and finally loss of viability becoming PI-positive. These transitional dose-dependent changes can be observed in bivariate plots as shown in Fig. 1a.

Considering the fraction of dead and live-apoptotic cells with oxCL (Q1 + Q3 in Fig. 1a), we quantified the cytotoxic potency using the median effect equation to compute the EC50 (Fig. 1b). We observed a similar dose-dependent reduction in MMP in 72 h samples (Fig. 1c). We observed increased MM (mean fluorescence intensity in Q2 and Q4) up to 10 μM ATO (Fig. 1d). Increased mitochondrial damage by ATO was associated to increased glucose uptake, as indicated by the probe 2-NBDG (Fig. 1e).

### CCCP collapsed MMP of BC-K562 cells in normoxia but decreased glucose uptake

**Fig. 2:**
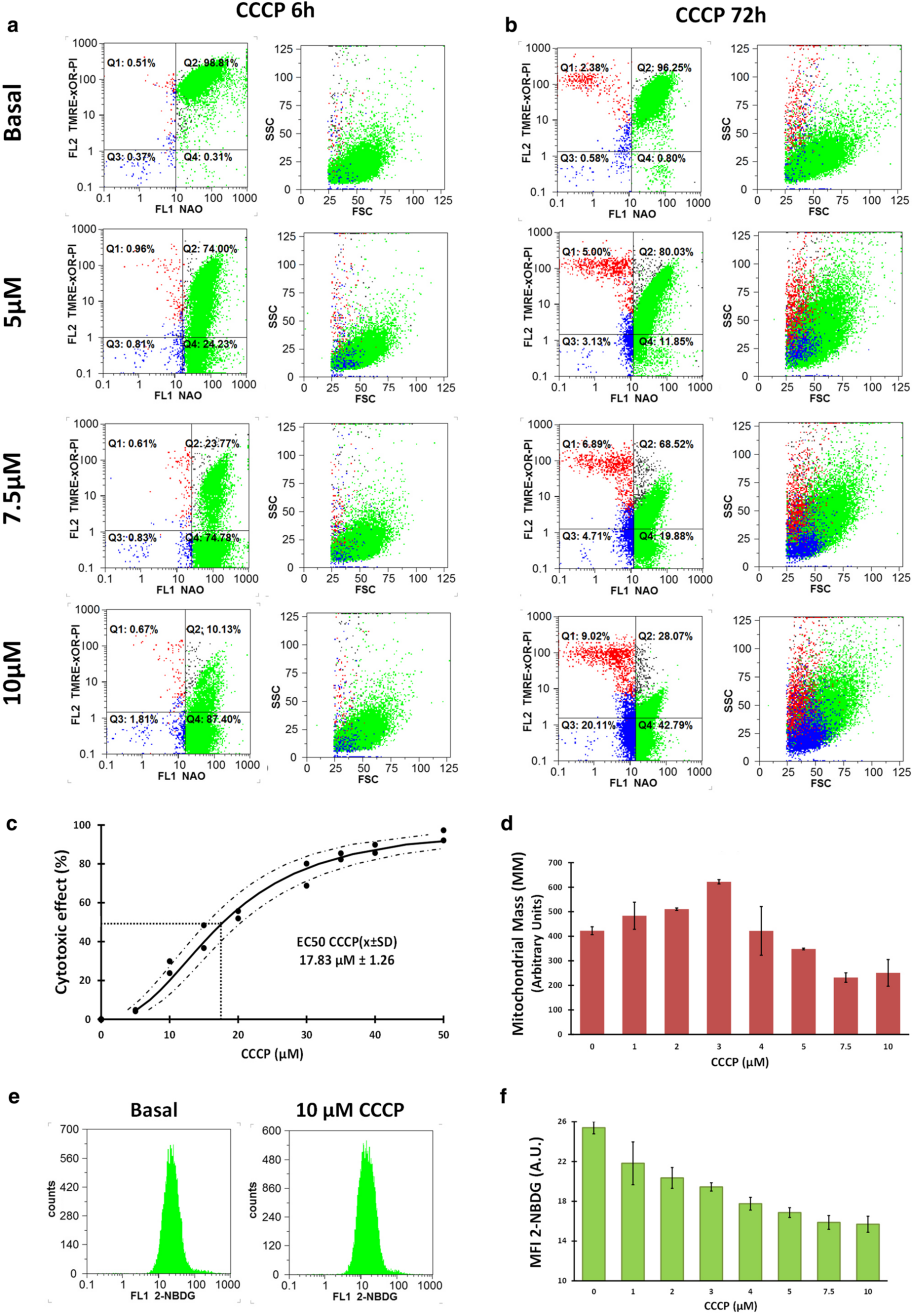
Cytotoxic effect of CCCP under normoxia in BC-K562 cells. **a** Collapse of MMP with rdCL at increasing doses of CCCP at 6 h treatment. **b** Partial restoration of MMP at 72 h with low apoptotic and dead figures (blue and red dots respectively) up to 10 μM CCCP. **c** Dose response curve obtained through regression using the median effect equation. Cytotoxic effect at each dose was computed from Q1 + Q3. **d** MM of cells at 72 h at increasing doses of CCCP. **e** Two representative histograms of cells labelled with 2-NBDG. **f** Bar graph showing decreased glucose uptake with increasing doses of CCCP. MFI: mean fluorescence intensity; A.U.: arbitrary units

The uncoupling of ETC by CCCP produced massive collapse of MMP at 6 h with conservation of rdCL (Fig. 2a) which was partially restored at 72 h depending on the dose of CCCP applied (Fig. 2b). Expanding the dose range and performing a regression using the median effect method, we obtained an EC50 of 17.83 for CCCP in normoxia (Fig. 2c). We further explored effects over the range 0-10 μM CCCP where a partial recovery of the MMP was observed at 72 h with high cell viability.

A large fraction of viable cells in samples treated with 5 μM CCCP and above, still showed high NAO fluorescence signal (rdCL) with collapsed-MMP (Q4 in Fig. 2b) after 72 h treatment. These cells were able to survive in spite of the restriction posed to OXPHOS by collapsed MMP and uncoupled ETC caused by CCCP. Therefore, cells survived with collapsed MMP and rdCL content, and even restored the MMP 72 h later (progressing from Q4 back to Q2, Fig. 2a, b).

An increased MM was observed at very low dose ranges (< 3 μM CCCP), while at higher dose ranges MM was reduced (Fig. 2d). Massive collapse of MMP restrains ETC and OXPHOS, and should force the activation of glycolysis as a compensatory source of ATP. However, we observed a progressive reduction in glucose uptake over the range 0-10 μM CCCP (Fig. 2e, f).

### CCCP rescued BC-K562 cells from hypoxia-induced death

**Fig. 3:**
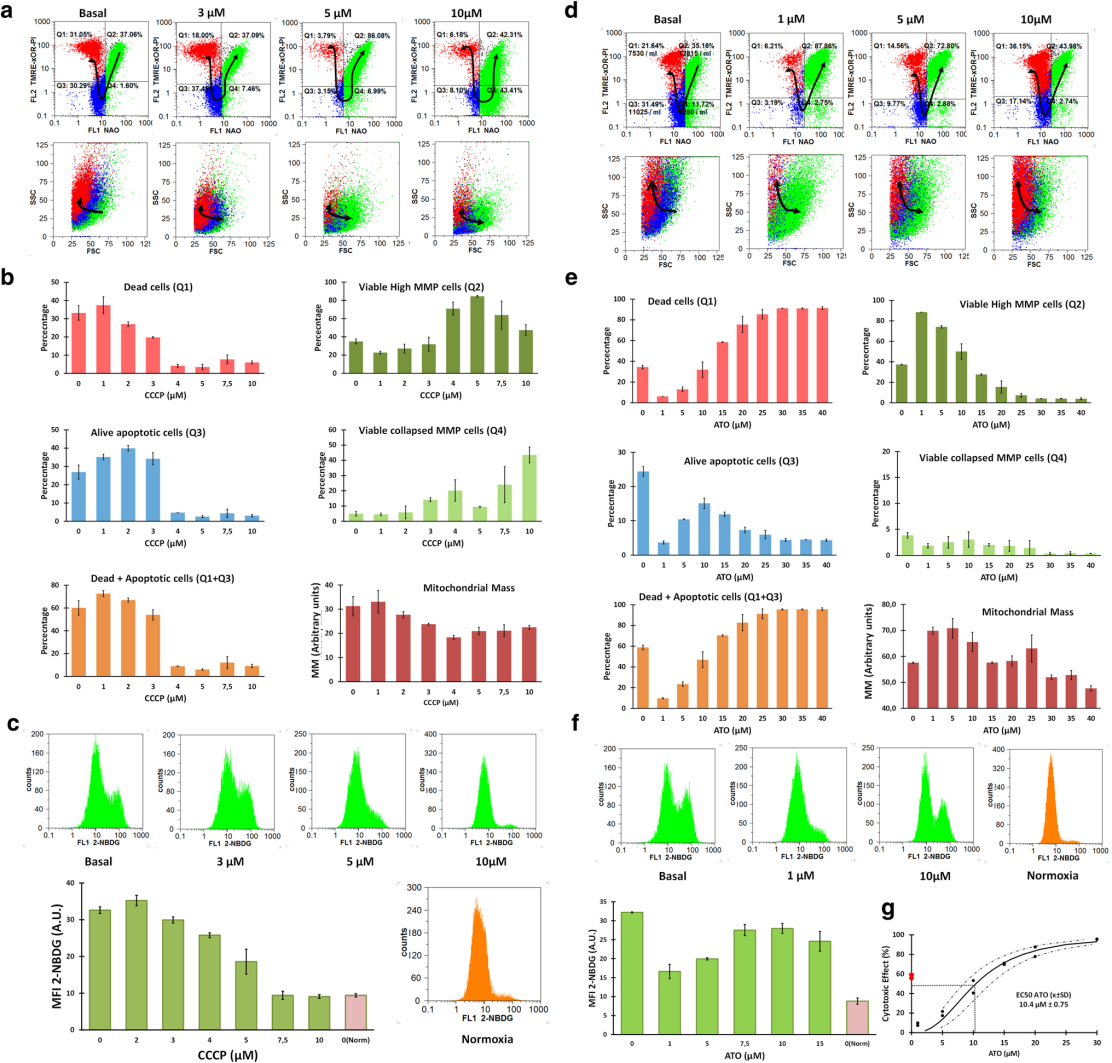
Cytotoxic effect of CCCP and ATO at 72 h under hypoxia in BC-K562 cells. **a** Representative dot plots of CCCP-treated cells under hypoxia. Dead and apoptotic cells (red and blue dots respectively) were about 60% in untreated cells but decreased with increasing CCCP dose, while live cells with rdCL (green dots) augmented, even showing collapsed MMP (Q4). **b** Bar graphs showing CCCP pro-survival effect with percentage of dead and apoptotic cells (Q1 and Q3 respectively), live cells with rdCL either with high MMP (Q2) or collapsed MMP (Q4), and MM changes with increasing CCCP dose. **c** Five representative histograms of cells labelled with 2-NBDG, and a bar graph showing decreased glucose uptake with increasing CCCP dose, approaching the same values observed in normoxia. MFI: mean fluorescence intensity; A.U. arbitrary units. **d** Representative dot plots of ATO-treated cells under hypoxia. Dead and apoptotic cells (red and blue dots respectively) decrease only at 1-5 μM ATO compared to untreated cells, and increased at higher ATO doses. **e** Bar graphs showing percentage of dead and apoptotic cells, live cells with rdCL and high MMP and MM changes with increasing ATO dose. Q1 + Q3 figures shows a biphasic shape with decline and rapid increases in cell death with ATO. **f** Four representative histograms of cells labelled with 2-NBDG, and a bar graph showing mostly high glucose uptake with increasing doses of ATO, close to that of untreated cells under hypoxia. MFI: mean fluorescence intensity; A.U. arbitrary units. **g** Regression dose–response curve using the median effect equation, with EC50 of ATO obtained at 72 h treatment. Red dots (untreated cells) show that ATO rescues cells from hypoxia below 5 μM and further kills cells at higher doses

Exposure to hypoxia for 72 h induced cell death in more than 50% of cells considering membrane damage and oxCL, and live cells in early apoptosis with oxCL (Q1 + Q3) (Fig. 3a, b). These figures were coincident to a previous study in BC-K562 cells under 1% O_2_, even though the authors used the method of AnnexinV with PI to quantify apoptosis [37]. Paradoxically, the mitochondrial damage induced by CCCP was beneficial for cell survival under hypoxia in a dose-dependent manner in the 0–10 μM range (Fig. 3a, b). For example, with 10 μM CCCP, there were 42% of viable cells with high MMP, and 43% of viable cells with collapsed MMP and rdCL, having normal morphology according to FSC and SSC (Fig. 3a). This means that more than 85% of cells were viable (Q2 + Q4), with a parallel decrease in early live-apoptotic cells (Q3) and dead cells (Q1) (Fig. 3a, b). The collapse of MMP with CCCP was either high, as it occurred at 10 μM, or low as with 5 μM, and yet in both situations the viability rose to about 90% (Fig. 3b). Therefore, MMP was needless for survival under hypoxia.

To complement this manual flow cytometry quadrant analysis using bivariate plots, we conducted an unsupervised classification through the FlowSOM algorithm [29], and we visualized the five dimensional phenotypic space (FSC, SSC, NAO, TMRE and PI) using tSNE plots [30]. This multidimensional analysis was in agreement with the manual analysis, and even detected higher values of live cells rescued from hypoxia-induced cell death by CCCP treatment over the 5–10 μM dose range. In addition, the study confirmed that rescued cells had either high MMP or low MMP, depending on the CCCP dose. At 5 μM CCCP dose, rescued cells showed the highest proportion in the high MMP group (matching Q2), while at 10 μM CCCP rescued cells were all in the low MMP group (matching Q4). In both cases rescued cells showed high NAO fluorescent signal (rdCL content). Early alive apoptotic cells matched the Q3 population in quadrant analysis, and were detected at 3 μM CCCP or below, almost disappearing at higher CCCP doses (Supp. Fig. S1)**.**

The MM was reduced within the dose range, where CCCP rescued BC-K562 cells from hypoxia-induced damage (Fig. 3b). Conversely, CCCP reduced glucose uptake within the dose range where cell survival was increased (4-10 μM), even approaching the baseline values observed in normoxia (Fig. 3c).

### ATO had a similar cytotoxic potency in normoxia and hypoxia

The dose–response curve of ATO and its EC50 under hypoxia was similar to the one obtained in normoxia (10.4 μM and 9.63 μM respectively) (Figs. 1b, 3g). However, we must consider that untreated cells had more than 50% death (red dots on the y-axis in Fig. 3g), meaning that at low doses ATO also had a rescuing effect (between 1 and 5 μM). Above EC50 cell death was increased in a dose-dependent manner (Fig. 3d, e, g). Contrasting CCCP results, there were no cells with collapse of MMP and rdCL (Q4) (Fig. 3e). Glucose uptake was reduced within the low dose range of ATO, although it attained the same uptake values of untreated cells at EC50 dose, well above the values observed in normoxia. (Fig. 3f).

### Inhibition of mitophagy with Mdivi1 or VCR increased MM and rescued BC-K562 cells from hypoxia-induced death

**Fig. 4:**
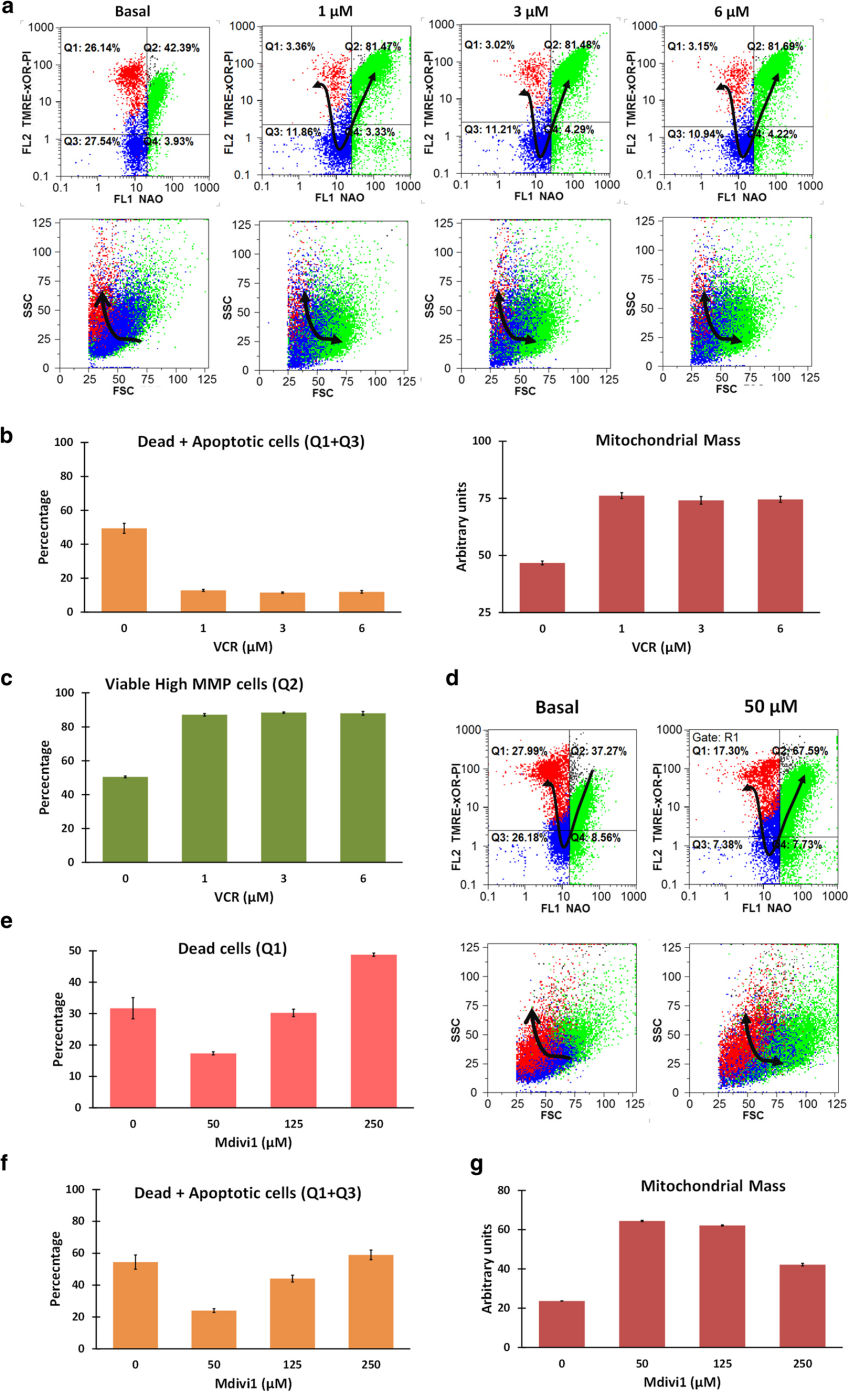
VCR and Mdivi1 rescued cells from hypoxia-induced death at 72 h in BC-K562 cells. **a** Representative dot plots of VCR-treated cells under hypoxia. Untreated cells show about 60% dead and apoptotic cells (red and blue dots respectively), but were rescued by 1–3 μM VCR, achieving more than 80% of live cells with rdCL and high MMP (green dots). **b** Bar graphs showing VCR pro-survival effect with percentage of dead and apoptotic cells, and increased MM in the VCR-rescued cells consistent with blockage of mitophagy. **c** Bar graphs showing increased MMP in VCR-rescued cells. **d** Representative dot plots of Mdivi1-treated cells under hypoxia, showing a rescuing effect from hypoxia-induced cell death at 50 μM Mdivi1. **e** Bar graphs showing that dead cells decreased at 50 μM Mdivi1 compared to untreated cells, but increased at higher Mdivi1 doses. **f** Dead + apoptotic cells also decreased at 50 μM Mdivi1 and increased at higher Mdivi1 doses. **g** Bar graphs showing increased MM in Mdivi1-treated samples, consistent with blockage of mitophagy and increased mitochondrial fusion

Low performing mitochondria are pruned by fission and cannot undergo further fusion, but are rather transported within mitophagosomes along microtubules to the lysosomes (mitophagy). Therefore, we blocked mitophagy upstream with the fission inhibitor Mdivi1, and downstream with the microtubules transport inhibitor VCR, assuming that blocking mitophagy would be detrimental for BC-K562 cell survival under hypoxia. Contrary to our expectations, both drugs rescued BC-K562 cells from hypoxia-induced cell death. Viability was increased up to 80% under hypoxia with VCR concentrations between 1 and 6 μM (Fig. 4a, b). VCR increased MM as expected from blocking the elimination of mitochondria (Fig. 4b). In addition, cells maintained high MMP (Fig. 4c). Blocking mitophagy with Mdivi1 rescued cells from hypoxia-induced death, with viability figures close to 70% (Fig. 4d). This effect was only observed below 50 μM and higher doses of Mdivi1 were cytotoxic (Fig. 4e, f). However, at 50 μM Mdivi1 there was a very large increase in MM, something that also negates the classical view that mitochondria must be eliminated to improve tolerance to hypoxia (Fig. 4g).

### VPA and DCA increased MM and rescued BC-K562 cells from hypoxia-induced death

**Fig. 5:**
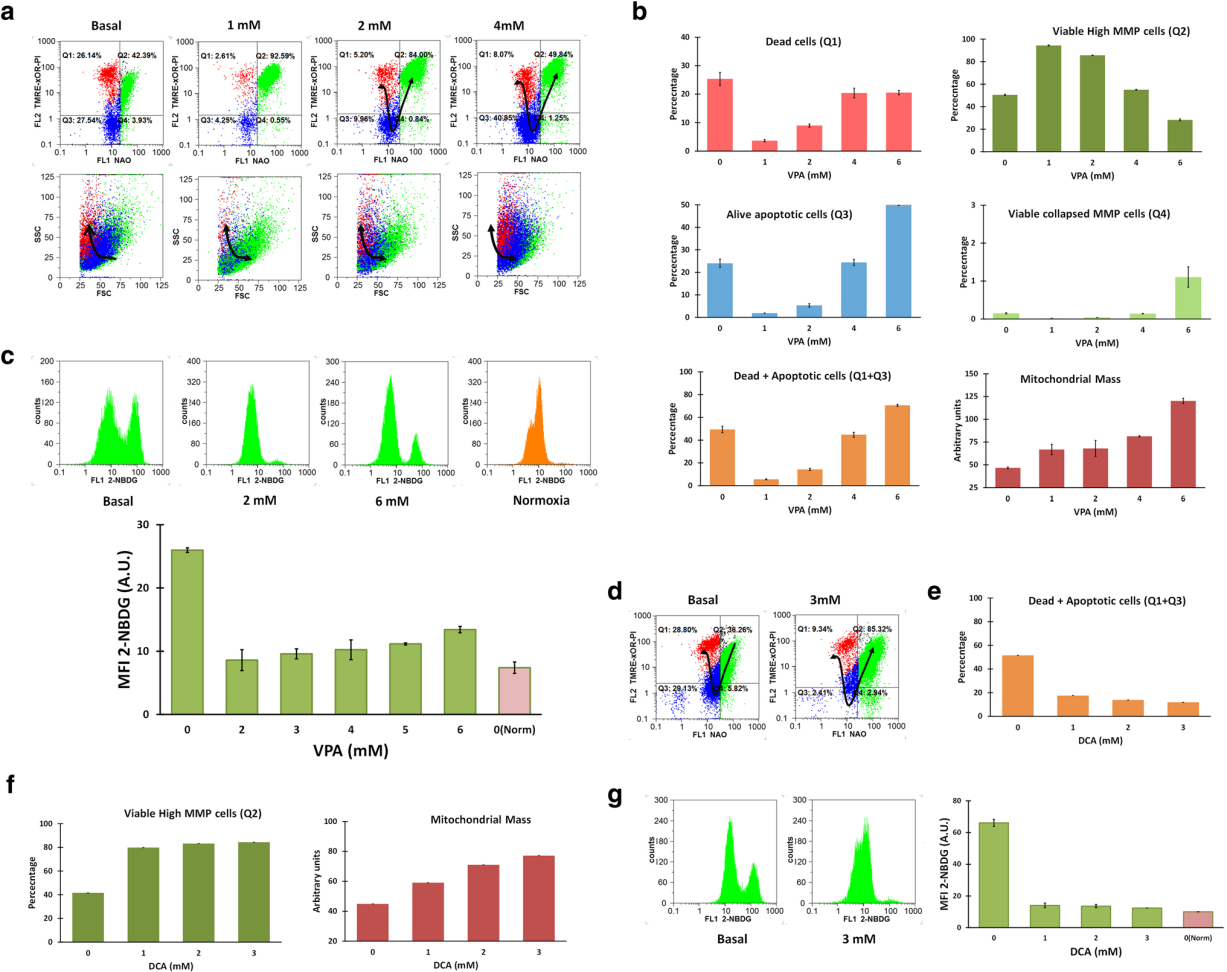
VPA and DCA rescued cells from hypoxia-induced death at 72 h in BC-K562 cells.** a** Representative dot plots of VPA-treated cells under hypoxia. Untreated cells show about 60% dead and apoptotic cells (red and blue dots respectively), but were rescued at 1–2 mM VPA, achieving above 80% of live cells with rdCL and high MMP (green dots). **b** Bar graphs showing VPA pro-survival effect with decreased percentage of dead and apoptotic cells, increased MMP and increased MM in the VPA-rescued cells consistent with increased mitochondrial biogenesis. VPA at 4 mM and above became cytotoxic. **c** Four representative histograms of cells labelled with 2-NBDG, and a bar graph showing strong decrease in glucose uptake within 1–6 mM VPA. MFI: mean fluorescence intensity; A.U. arbitrary units. **d** Representative dot plots of DCA-treated cells under hypoxia showing a rescuing effect from hypoxia-induced cell death at 3 mM DCA. **e** Bar graphs showing a strong rescuing effect from hypoxia-induced cell death at 1–3 mM DCA from about 60% in untreated cells to less than 10% in DCA treated cells. **f** Representative histograms of cells labelled with 2-NBDG, and a bar graph showing strong decrease in glucose uptake within 1–3 mM DCA. MFI: mean fluorescence intensity; A.U. arbitrary units

VPA induces genes involved in mitochondrial biogenesis (PGC1α, TFAM) and we assumed VPA-treatment of BC-K562 cells would be deleterious to survival under hypoxia. However, VPA rescued cells from hypoxia-induced death, particularly within the dose range 1–2 mM, with figures even above 90% of viability (Fig. 5a, b). The cells showed high MMP and high MM (Fig. 5b). On the other hand, this increase in MM under hypoxia was paradoxically associated to a decrease in glucose uptake in VPA-treated cells compared to untreated cells. Even at high doses of VPA with a large increase in MM and decreased survival rates, glucose uptake remained low (Fig. 5c). This result suggested a connection between high MM and alternative metabolic sources to sustain glycolysis, such as biosynthetic metabolic pathways that intersect at the mitochondria.

DCA inhibits PDK1 and increases PDH activity. Therefore, it fosters entrance of pyruvate to the Krebs cycle and formation of acetyl-CoA and citrate. This effect is opposite to the action of HIF that increases expression of PDK1 and blocks PDH. By improving Krebs cycle and OXPHOS, DCA is considered a drug with an "anti-Warburg effect" that would be highly toxic under hypoxia.

However, BC-K562 were rescued from hypoxia-induced death by 1–3 mM DCA with viability above 80% (Fig. 5d, e, f). Unexpectedly, there was a dose-dependent increase in MM. Therefore, DCA had two HIF-opposing effects: increased MM instead of reduction by mitophagy, and increased PDH activity, yet these effects resulted in rescue of cells from hypoxia-induced death. Moreover, DCA decreased glucose uptake (Fig. 5g), again pointing to mitochondrial anabolic pathways supporting glycolysis in an unconventional form. The beneficial effect of increased MM by 3 mM DCA appeared to be independent of OXPHOS, since it was completely preserved when combined to CCCP over a range 1–10 μM (Supp. Fig. S2). Contrasting the effect of single drugs, the VPA + VCR combination was synergistic and highly cytotoxic for survival under hypoxia (Supp. Fig. S3a, b). We observed very large dose reduction indexes, especially for VCR. Therefore, blockage of mitophagy with increased mitochondrial biogenesis appeared to break the delicate balance between mitochondrial quality and quantity.

### Genes differentially expressed in BC-K562 cells under hypoxia

All drugs tested had paradoxical responses under hypoxia, with most of them having patent rescuing effects from hypoxia-induced death instead of cytotoxicity. Since these drugs target mitochondria or MRCSR, paradoxical effects could be related to hypoxia-induced metabolic changes. In a recent study, Jain et al., exposed BC-K562 to 1% O_2_ and 21% O_2_ during 72 h, performed RNAseq on three samples under each condition, and deposited raw data in the curated GEO database (GSE144527) [32]. Since experimental conditions and time of exposure matched our own study with BC-K562 cells, we used this RNAseq dataset to explore MP changes induced by hypoxia from a gene expression perspective.

The analysis of DEG through the R package limma, showed significantly increased expression of genes involved in HIF stabilization (P4HA1, P4HB), mitophagy (BNIP3, BNIP3L, SQSTM1, ATG13, MAP1LC3B), increased glucose uptake (SLC2A3/GLUT3), regulation of glycolysis and pentose phosphate pathway (PKM1/2, LDHA, HK2), cholesterol synthesis (HMGT, HMGCS1, MVN), and fatty acid synthesis (FASN). The malic enzyme (ME), and the anti-apoptotic protein MCL1 were also upregulated, while many genes involved in anti-oxidant response were down regulated under hypoxia including CAT, PRDX1, PRDX2, PRDX4, and GSS. Also, MFN2 and OPA1, involved in mitochondrial fusion, were downregulated under hypoxia (Supp. Table S1). Analysis through Gene Ontology (GO) knowledge-base, conducted using the GOANA function of limma R package, showed GO terms with significantly DEG under hypoxia that were associated to mitochondrial cellular components, metabolism-related biological processes and functions (Supp. Table S2). The analysis using Kegg database through KEGGA function of limma R package, showed significantly DEG in pathways like glycolysis and gluconeogenesis, NADH regeneration, HIF-1 signalling, autophagy and steroid biosynthesis among others (Supp. Table S3).

**Fig. 6:**
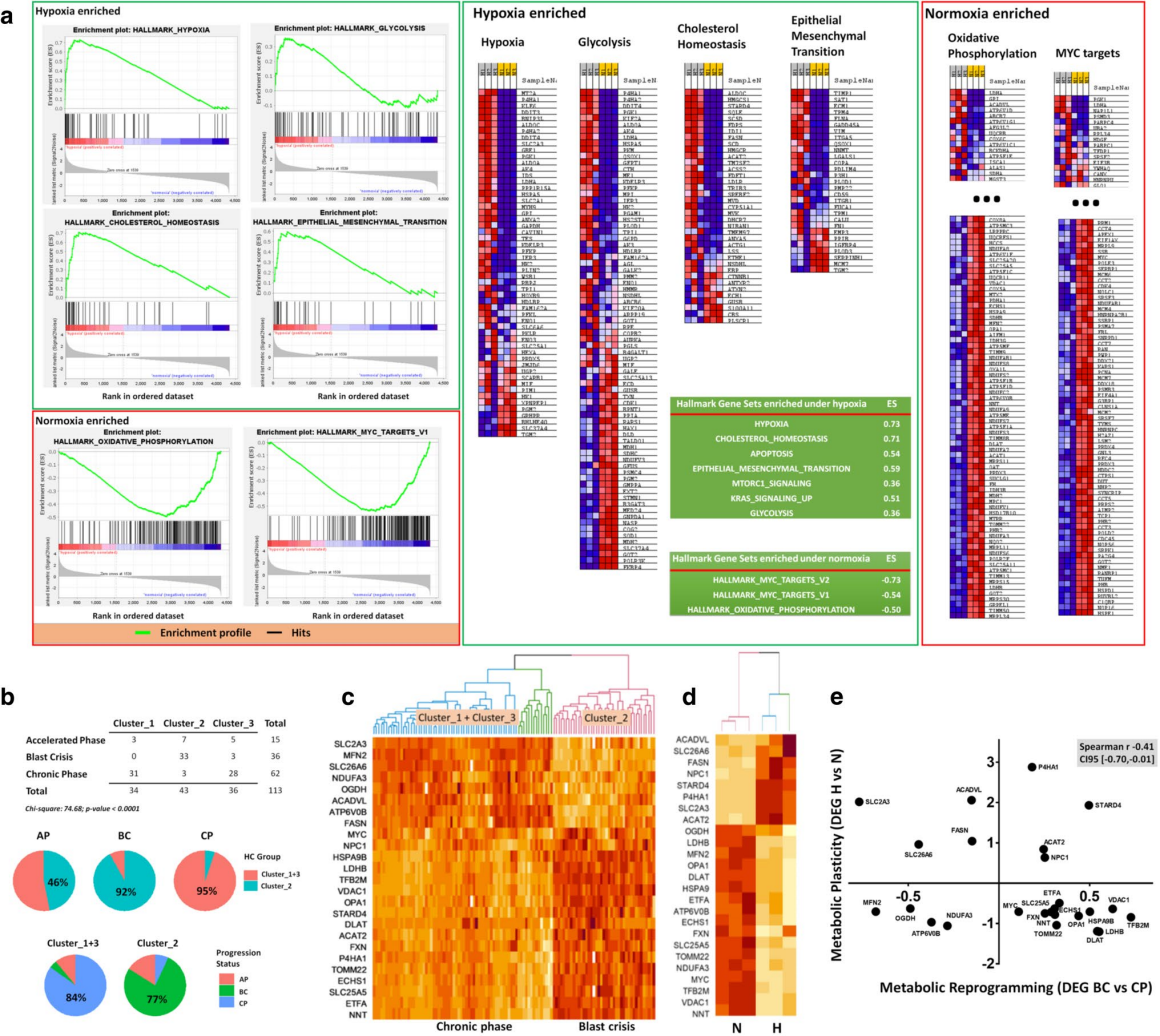
A 25-gene metabolic plasticity signature derived from BC-K562 cells under hypoxia. **a** Gene set enrichment analysis of BC-K562 cells exposed to 1% hypoxia for 72 h. The RNA expression values of 4362 genes of three replicates under hypoxia and three replicates under normoxia were compared over 50 functional Hallmark gene lists (MSigDB). Enrichment, indicating RNA over or under-expression, is represented by profile line graphs, where the black bar represents a single gene from the list of 4362 genes, which matched a gene of the Hallmark list (hit), and the line represents over-expression (left peak) or under-expression (right peak) in a comparison hypoxia vs normoxia, or normoxia vs hypoxia (hypoxia-enriched, and normoxia-enriched respectively). Clustered black bars on the left indicate over-expression, and clustered black bars on the right indicate under-expression. The enrichment score (ES) provides a magnitude of over-expression (positive value) or under-expression (negative value) of genes of the Hallmark functions (see Materials and Methods section). The detailed genes (hits) found in each Hallmark gene list are shown as heatmaps on the right (they correspond to the black bars in the enrichment profile graphs shown on the left). Red squares are genes over-expressed and blue squares are gene under-expressed in hypoxia. Each column of the heatmaps represents an RNAseq sample. Since the middle part shows genes expressed similarly in hypoxia and normoxia we omitted the middle part (indicated by three large dots). **b** Contingency table exploring association between clinical categories CP, AP and BC and unsupervised classification of 113 CML patients according to their RNA expression profile. The patients were classified into Cluster_1, Cluster_2 and Cluster_3, considering only RNA expression values of a 25-gene metabolic plasticity signature, selected among those genes that we found differentially expressed in BC-K562 cells under hypoxia. Pie charts illustrates how the three clinical categories fit in two main “metabolic” clusters (Cluster_1 + 3 and Cluster_2) with CP and BC cases clearly separated, and AP cases fitting half in each cluster. **c** Heatmap showing expression profile with unsupervised classification of 113 CML patients using the 25-gene metabolic plasticity signature obtained from BC-K562 cells under hypoxia. The 25 genes are listed on the left of the heatmap. BC patients are clearly associated to the profile of cluster_2 while CP patients are associated to profiles of Cluster_1 + 3. **d** Heatmap presenting unsupervised classification of six BC-K562 transcriptomes and showing that the 25-gene MP-signature also discriminates between BC-K562 cells under hypoxia and normoxia. **e** Correlation plot showing that the gene expression profile of these 25 genes is different and inversely correlated when comparing BC patients to CP patients (metabolic reprogramming) than when comparing BC-K562 cells under hypoxia vs normoxia (metabolic plasticity).

Gene set enrichment analysis (GSEA) showed significantly enriched Hallmark gene sets under hypoxia that included “Hypoxia response” (p < 0.001; FDR < 0.001), “Glycolysis” (p < 0.001; FDR < 0.007), “Cholesterol Homeostasis” (p < 0.001; FDR < 0.001), “Apoptosis” (p < 0.001; FDR < 0.001), “MTORC1 signalling” (p < 0.001; FDR < 0.002), “KRAS signalling UP” (p < 0.008; FDR < 0.004), and “Epithelial mesenchymal transition” (p < 0.001; FDR < 0.001) (Supp. Table S4, Fig. 6a). Conversely, among significantly enriched Hallmark gene sets under normoxia we noticed “MYC targets” (p < 0.001; FDR < 0.001) and “Oxidative Phosphorylation” (p < 0.001; FDR < 0.001) (Supp. Table S5, Fig. 6a).

### A metabolic plasticity signature derived from DEG and GSEA analysis

Considering leading edge genes from GSEA and significantly DEG (Fig. 6a, Supp. Tables S1-Table S3), we chose 99 genes as a potential signature that appeared relevant to MP under hypoxia, including genes involved in OXPHOS and ETC, Krebs cycle, cholesterol synthesis, fatty acid synthesis, mitochondrial fusion, mitophagy, glycolysis, pentose phosphate pathway, antioxidant response, anti-apoptosis and alternative sources of biosynthetic precursors (Supp. Table S6). This MP-signature clearly discriminated between BC-K562 cells under hypoxia and normoxia (Supp. Fig. S4a, Table S7).

### Evaluation of the MP-signature in a transcriptomic dataset from CML patients

We further explored if this MP-signature could also discriminate between BC-CML patients and CP-CML patients, to ascertain if genes involved in MR along the disease progression, could match genes involved in MP (correspondence between the two metabolic dimensions). To this end, we explored the MP-signature among cells from CML patients samples in BC, AP, and CP, by using the GSE4170 microarray dataset. This dataset consisted of transcriptomes from a large CML patient’s cohort [33].

We conducted unsupervised classification of 113 patients based on their MP signature through hierarchical clustering, and we evaluated the first three most different groups. BC samples were classified mostly in one group, CP samples were classified in the two other groups, while AP samples were distributed among all three groups, but mainly with BC samples (Supp. Fig. S4b, c). Therefore, the MP-signature identified BC patients as a separated group, making evident a correspondence between MR and MP in the BC phenotype. The association between MP-signature category and clinical category was highly significant (p < 0.0001). When we next restricted the 99 gene set to a smaller signature of 25 genes (Supp. Table S8), 92% of BC cases corresponded to a single cluster (Cluster_2), and 95% CP cases corresponded to the other two clusters (Cluster_1 + 3). (Fig. 6b, c). About 46% of AP cases were in Cluster_2 together with BC cases, while the 53% remaining were in Cluster_1 + 3 with CP cases. Cluster_2 consisted of 77% BC cases and 16% AP cases (93% both), while Cluster_1 + 3 consisted of 84% CP cases (Fig. 6b, c). Noticeably, OXPHOS-related genes that were down-regulated under hypoxia in BC-K562 cells, appeared characteristically up-regulated in BC patients compared to CP patients. By contrast, genes of cholesterol metabolism were up-regulated under hypoxia in BC-K562 cells, but were down-regulated when comparing BC samples to CP samples (Fig. 6c). This MP-signature made of 25 genes also distinguished BC-K562 cells under normoxia and hypoxia considering the 6 samples from GSE144527 (Fig. 6d). It is noticeably that most of these genes were unrelated to HIF-targets. The differential expression of many genes was dissimilar in the comparison between CP and BC (metabolic reprogramming), and the comparison between normoxia and hypoxia (metabolic plasticity). For example MFN2 was down-regulated in BC, and OPA1 was up-regulated in BC compared to CP. However, both MFN2 and OPA1 were down-regulated in BC-K562 cells under hypoxia compared to the same cells under normoxia. Therefore, the gene set that distinguished BC from CP was the same as the gene set that distinguished hypoxia from normoxia, but the sense and magnitude of differential gene expression in both situations was different. (Fig. 6c, d and Supp. Fig. S5). In fact there was an inverse correlation between differential expressions BC vs CP compared to hypoxia vs normoxia (Spearman rank correlation coefficient − 0.41; 95% CI [− 0.70,− 0.01], p < 0.05), (Fig. 6e). Therefore, the expression profile of this 25-gene set may reflect changes due to both MR and MP of blast crisis cells.

## Discussion

In this work we explored the cytotoxic potency of drugs that target mitochondria and MRCSR under < 1% hypoxia during 72 h in BC-K562 cells. Less than 50% of untreated cells survive under these conditions. These figures are in complete agreement with a previous study that quantified dead and apoptotic cells at 72 h and 1% O_2_ [37]. In addition, further studies showed that surviving untreated-BC-K562 cells acquire traits of leukaemia stem cells at day seven, and even decrease Bcr-Abl expression at the protein level [38]. Paradoxically, many of the mitochondria-targeted drugs that we tested, rescued cells from hypoxia-induced death at 72 h instead of killing them. Since hypoxia represented a sudden environmental change driving rapid metabolic changes, we inferred that the cause of the profoundly altered cytotoxicity was linked to these changes, particularly those linked to mitochondrial functions, since mitochondria initiates apoptosis through MOMP. Mitochondria are not a significant source of ATP under hypoxia, and HIF-mediated response to hypoxia involves its elimination through mitophagy [10]. However, mitochondria may not be superfluous under hypoxia in BC cells, considering that abnormal anabolic pathways may interweave at the mitochondria [39]. The gene expression analysis of BC-K562 supports this idea, denoting high enrichment of gene sets associated to fatty acid synthesis and cholesterol homeostasis. These anabolic routes require a strong support of pathways operating at the mitochondria, such as citrate efflux and truncated forms of the Krebs cycle. How these anabolic rather than bioenergetic functions of mitochondria impact on apoptosis threshold is only partially known, and several hypothesis have been raised. Among these evidences are increased anti-apoptotic proteins such as MCL1, low performing ETC that is less prone to MOMP, and a stronger antioxidant capacity provided by large amounts of NADPH.

Cancer cells have several changes in metabolic pathways that increase along disease progression. These MR changes are considered to progress as waves of gene expression changes, occurring along extended periods of time [40]. However, cell metabolism is dependent on the microenvironment, and environmental conditions of cancer are not stable but rather often subject to sudden changes. Therefore, cancer cells may not only have an abnormal metabolism, but have also the capability of transiently changing the metabolic program [5]. This second dimension of transient metabolic changes are known as MP [41]. We inferred that MP would be greater in advanced disease, providing BC cells a faster metabolic adaptation to transient environmental changes such as hypoxia and lack of nutrients [42].

Our hypothesis was that the cause of paradoxical response to drugs under hypoxia could be related to metabolic changes of cells that could impact on drug targets. For example, ETC becoming disposable for ATP synthesis under hypoxia, but Krebs cycle still operating to support anabolic pathways, could transform a drug vulnerability under normoxia to a drug-induced growth advantage under hypoxia. To explore if such changes effectively occur, we compared the transcriptomes of BC-K562 cells under normoxia and hypoxia, using raw data from an RNAseq dataset that became available recently in GEO database (GSE144527) [32].

Our analysis of GSE144527 allowed us to define a MP-signature of 25 genes related to mitochondrial function, mitophagy, mitochondrial fusion, Krebs cycle enzymes, glycolysis, fatty acid synthesis, and cholesterol metabolisms that were differentially expressed in BC-K562 after 72 h under 1% hypoxia. This MP-signature was sufficient to completely discriminate cells under normoxia and hypoxia through unsupervised classification, confirming that the occurrence of these metabolic changes was a distinctive feature of hypoxia-adaptation of cells, and could potentially impact on drug targets. Noticeably, this MP-signature had only a minor representation of classical HIF target genes. This shows how critical and archetypal is this expression profile in hypoxia adaptation of BC-K562 cells.

In agreement with a prominent role of mitochondrial genes under hypoxia, we found that increased MM was associated with rescue from hypoxia-induced death. This increase resulted from very dissimilar grounds such as blocking mitophagy (VCR and Mdivi1), increased mitochondrial biogenesis (VPA), and even increased MM by DCA probably owing to targeting PGC1α as off-target, apart from its main action increasing PDH activity (we provide the analysis of a microarray dataset of BC-K562 cells treated with 1 mM VPA that was available from GEO database in Supp. Fig. S6a).

The exception was CCCP that decreased MM while rescuing cells from hypoxia-induced cell death. This may be due to its massive induction of mitophagy. In fact, CCCP is used as a positive control of mitophagy in experimental settings [43]. The beneficial effect of increased MM was not due to augmented ETC and OXPHOS since this is hampered by low O_2_. In fact, CCCP caused rescue from hypoxia-induced death while completely preventing the function of ETC.

**Fig. 7:**
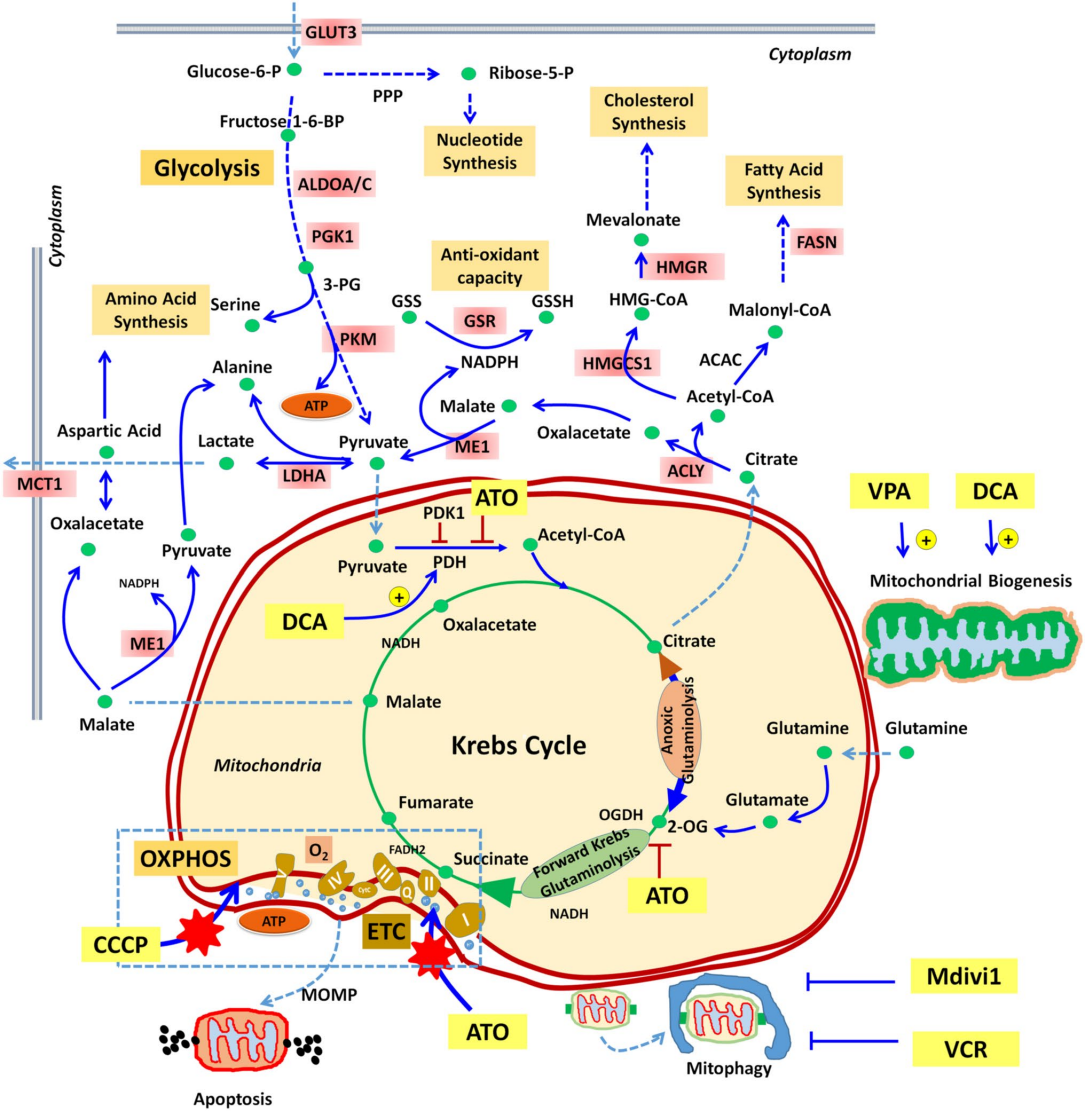
A simplified diagram representing some relevant anabolic and catabolic pathways intersecting at mitochondria that may be relevant in metabolic plasticity of BC-K562 cells and the paradoxical effects of drugs under hypoxia. The Krebs cycle can operate as a source of biosynthetic precursors through cataplerotic pathways such as citrate efflux, but must be compensated by anaplerotic pathways such as glutaminolysis as a source of 2-oxoglutarate (2-OG). A truncated Krebs cycle may operate in reverse sense even under complete absence of oxygen (anoxic glutaminolysis), or may operate in forward sense, generating FADH from succinate at ETC complex II, even with very low oxygen (forward Krebs glutaminolysis). However, forward Krebs glutaminolysis coupled to citrate efflux requires continuous supply of acetyl-CoA. This is inhibited by ATO and improved by DCA. In addition, ATO partially inhibits 2-oxoglutarate-dehydogenase (2-OGDH). ROS-mediated damage to the ETC by ATO and CCCP may be reduced under lack of oxygen. The graph shows in light pink some genes significantly upregulated under hypoxia compared to normoxia (p-value < 0.05) in untreated BC-K562 cells (GSE144527), and how citrate efflux appears coupled to cholesterol and fatty acid synthesis. PPP pentose phosphate pathway, MCT1 monocarboxylate transporter1 /SLC16A1, HMG-CoA hydroxy-methyl-glutaryl-CoA, and the remaining are conventional gene symbols (https://www.genecards.org)

Moreover, CCCP + DCA rescued cells with complete collapse of MMP and increased MM (particularly at the highest dose of 10 μM CCCP), enforcing the idea that the beneficial effect of increased MM was other than ETC and OXPHOS (Supp. Fig. S2). It is important to emphasize that collapse of MMP with rdCL content (high NAO fluorescence signal) does not indicate mitochondrial damage, but just cells where there is no possibility of OXPHOS, because without MMP the ETC becomes non-functional. On the other side, CCCP does not compromise the Krebs cycle and allows other mitochondrial anabolic pathways to continue fulfilling their functions. The anabolic functions of mitochondria involve an active Krebs cycle with cataplerotic pathways such as citrate or malate efflux, inter-conversion with amino acids such as aspartic acid (oxaloacetate) and alanine (pyruvate), and continuous replenishment through anaplerotic pathways such as glutaminolysis (Fig. 7) [3]. The increase in PDH activity as caused by DCA fosters citrate formation from acetylCoA and oxalacetate. Therefore, the outstanding pro-survival effect of DCA can be linked to citrate efflux and support of biosynthetic pathways in the cytoplasm, such as cholesterol homeostasis and fatty acid synthesis, with high anti-oxidant capacity provided by increased NADPH (Fig. 7). In contrast to DCA, ATO inhibits PDH and 2OGDH [17]. These two enzymes are critical for Krebs cycle, even when it operates only in support of anabolic pathways. Therefore, ATO targets the core of both bioenergetic and anabolic functions of mitochondria (we provide the analysis of a microarray dataset of BC-K562 cells treated with 1.5 μM ATO that was available from GEO database in Supp. Fig. S6b). Krebs cycle is required for anoxic glutaminolysis, where citrate is formed by reductive carboxylation (reverse and truncated Krebs cycle), and is also required in forward-glutaminolysis where a truncated Krebs cycle is coupled to ETC complex III through succinate, and allows citrate efflux by combination of oxalacetate and acetyl CoA (Fig. 7) [3, 44, 45]. Therefore, it is not surprising that ATO was the only toxic drug in hypoxia for BC-K562 cells, hampering all forms of mitochondrial functions. In addition, increased MM with lack of mitochondrial quality control provided by blockage of mitophagy, was highly toxic showing exceedingly high synergism and dose reduction indexes (Supp. Fig. S3). This result implies that the benefit provided by increased MM is abruptly offset beyond a certain level of accumulated mitochondrial defects.

Noticeably, survival from hypoxia-induced death was associated not only with increased MM but with decreased glucose uptake compared to untreated cells, and even approaching values comparable to normoxia. This is a remarkable result, since ATP must be provided by increased glycolysis under hypoxia. The DEG and GSEA analysis confirmed increased glycolysis in untreated cells. However, we observed a consistent decrease of glucose uptake in drug-rescued surviving cells. This suggests that glycolysis may be somehow aided by anabolic pathways fostered by mitochondria through processes such as citrate efflux (Fig. 7) [3]. After GU and conversion of glucose into glucose-6-phosphate (G6P), glucose carbons may take a number of alternative downstream routes, including glycolysis and ATP production, and diversion into the PPP pathway for the supply of biosynthetic precursors and NADPH [46]. It is possible that increased availability of mitochondria-derived precursor metabolites for biosynthesis processes could decrease the need for routing of G6P to PPP, increasing the availability for the glycolysis pathway. In this way, the demand for GU and conversion to G6P could be decreased to some extent. The regulation of these metabolic pathways appears highly dynamic and complex, and it is far from being understood in cancer cells, although some studies propose a role for isoforms of PKM in controlling the balance between G6P routed to PPP and to glycolysis according to the bioenergetic and biosynthetic demands [47, 48]. In this sense, we could speculate that cytoplasmic and mitochondrial metabolic pathways would be coordinated in such a way that, the more mitochondria supporting anabolic roles, the less the demand for GU given a certain level of demand for bioenergetic and biosynthesis processes.

The paradoxical effects of mitochondria-targeted drugs with increased MM and decreased glucose uptake occurred in cells that without treatment showed changes in the 25-gene MP signature. Since MP represents a transient adaptation driven by the environment, we asked if these same gene set could work as a MR-signature in samples from CML patients. Blast crisis cells showed a different expression profile in this 25-gene set, and this MR-signature discriminated between samples from BC patients and CP patients. Therefore, the expression profile of the same gene set could capture changes between hypoxia and normoxia, and changes between CP and BC patients. However, this profile in BC cells was different in both comparisons. In fact, the expressions of these genes were inversely correlated. Genes that were downregulated in response to hypoxic environment, were often those upregulated in BC patients compared to cells in CP patients. This again underscores that MR is an average profile correlated to progression, and that expression changes may occur in a second metabolic dimension driven by environment (plasticity) that may involve not only O_2_, but availability of nutrients such as glucose and amino acids [37]. Therefore, methods comparing two environments can unveil this second metabolic dimension and provide a better characterization of altered metabolism in advanced disease.

In a previous work, we found no correlation to disease progression of drug potencies in a single environment (normoxia or hypoxia), but a strong correlation to differential response of drugs targeting MRCSR between normoxia and hypoxia in CLL patients [25]. Here we substantiate those findings through gene expression profile, showing that an environment-driven differential signature was also highly correlated to progression in CML. We conclude that both MP and MR may impact drug potency. Understanding the interconnection of MP and MR with drug potency can give hints to overcome resistance. Recent studies have shown several successful examples including Venetoclax and ATO, TKIs and ATO, Venetoclax and CB839, and inhibitors of fatty acid oxidation such as Etomoxir, highlighting that the Krebs cycle appears as a relevant target to suppress anabolic functions of mitochondria [49–52].

## Supplementary Information

Additional file 1 Fig. S1Unsupervised classification through flow cytometry self-organized maps (FlowSOM) algorithm, and visualization of the five dimensional space (FSC, SSC, NAO, TMRE-XOR-PI, and PI) through tSNE plots was in agreement with the manual quadrant analysis. a) Visualization of the five-dimensional phenotypic space (FSC, SSC, NAO, TMRE-XOR-PI, and PI) of CCCP-treated BC-K562 under hypoxia through tSNE plots. Samples corresponding to untreated cells and CCCP-treated cells with 1, 3, 5 and 10 μM CCCP were combined in a single dataset. Each point represents a single cell in the five-dimensional space. Similar cells are close in the two-dimensional t-SNE plot, while distant clusters correspond to phenotypically different groups of cells. A third parameter is represented as a heatmap colour code (NAO, TMRE-xOR-PI, PI, FSC, and SSC) to identify what population represents each cluster (annotation). Cells with high NAO (rdCL) were split in two groups with high and low MMP. Live cells and dead cells as indicated by PI content were dissimilar (distant), and a smaller transitional group of live cells was observed in between both large groups (arrow). b) Visualization of cells corresponding to untreated samples and CCCP-treated samples in the tSNE plot. The rescuing effect from hypoxia-induced death at increasing doses of CCCP is show as a transition from the “dead” cells cluster to the live cells clusters, by representing only cells from each sample at a time. At 5 μM CCCP the high MMP cluster is the larger cluster, while at 10 μM CCCP, the low MMP cluster is the largest (red arrows). In both cases the clusters corresponded to live cells with high NAO signal (rdCL) as shown in panel a). c) Unsupervised classification of the same dataset of cells shown in panels a) and b) conducted through the FlowSOM algorithm using five parameters (FSC, SSC, NAO, TMRE-XOR-PI, and PI). The algorithm identified 100 groups of very similar cells (very close in the five-dimensional space), and represented these 100 groups in a two-dimensional tree-like plot. Again, closer circles correspond to phenotypically similar groups. In addition the FlowSOM algorithm identified 8 metaclusters (higher order clusters built by aggregating similar “circle” clusters). Each of the circles shows a pie chart symbol with expression values of FSC, SSC, NAO, TMRE-XOR-PI, and PI that allows the identification (annotation) of the metaclusters. As indicated by the labels, the main metaclusters were: dead cells (Population 6), live cells with high MMP (Population 0), live cells with low MMP (Population 1), and a small transitional metacluster of early live apoptotic cells having low NAO signal (oxCL) apart from low MMP (Population 4), which fitted between dead cell and both main metaclusters of live cells. d) The unsupervised FlowSOM analysis showed a result matching the manual analysis by quadrants. The tSNE plot on the left shows the location in the five dimensional space of the four main populations identified by FlowSOM. The tSNE plot on the right shows the location in the five dimensional space of the four populations identified through manual quadrant analysis. Population 0 matched Q2 (live cells with rdCL and high MMP), population 1 matched Q4 (live cells with rdCL and low MMP), population 6 matched Q1 (dead cells), and population 4 only partially matched Q3 (early live apoptotic cells). The manual analysis was more restrictive than the FlowSOM algorithm in the classification of cells as non-apoptotic (Q4 was smaller than population 1 as noted by light brown dots, and Q3 was larger than population 4 as noted by green dots). (JPG 2662 KB)

Additional file 2 Fig. 2. DCA combined with CCCP rescued cells from hypoxia-induced death at 72 h in BC-K562 cells. a) Representative dot plots of 3 mM DCA + 5 μM CCCP-treated cells under hypoxia. Untreated cells show about 60% dead and apoptotic cells (red and blue dots respectively), but were rescued by combined drugs. b) Bar graph showing pro-survival effect of 3 mM DCA combined with all doses of CCCP tested (0-10 μM CCCP) under hypoxia, achieving less than10% dead + apoptotic (Q1 + Q3). c) Bar graphs showing increased MM in rescued cells with combined DCA + CCCP. About 40% of rescued cells had collapsed MMP with 10 μM CCCP + 3 mM DCA. d) Two representative histograms of cells labelled with 2-NBDG, and a bar graph showing strong decrease in glucose uptake within 3 mM DCA combined with all CCCP doses tested (0-10 μM CCCP), in all cases approaching values comparable to normoxia. MFI: mean fluorescence intensity; A.U. arbitrary units. (JPG 654 KB)

Additional file 3 Fig. 3. VPA and VCR are synergic and highly cytotoxic against BC-K562 cells when combined under hypoxia. a) Representative dot plots of single and combined VPA and VCR treatment of BC-K562 cells under hypoxia. Combined treatment has no rescuing effect from hypoxia-induced death and appears highly cytotoxic. b) VPA and VCR were highly synergic with combination index (CI) well below 1.0 along the entire effect range and a value of 0.30 at 50% effect level (CI50). Assessment of interaction through CI method: For each cytotoxic level i, the combination index (CI) was calculated as: CI (i) = Dac(i) /Das(i) + Dbc(i) /Dbs(i), where Dac(i) and Dbc(i) are the doses of drugs a and b respectively required in the combination a + b to produce an effect level i. Das(i) and Dbs(i) are the doses of drug a and b respectively, required to produce an effect level i when used as single drugs. c) Drug reduction index (DRI) plot showing the increase in potency particularly of VCR with a value of 712 at 50% cytotoxic effect (DRI50). Assessment of interaction through DRI method: For each cytotoxic level i, the dose reduction index (DRI) for drugs a and b DRIa(i) and DRIb(i) were calculated as: DRIa(i) = Das(i) /Dac(i) and DRIb(i) = Dbs(i) /Dbc(i). Calculations were performed with the software Calcusyn (Biosoft, UK), which implements the above formulas [25, 31] (JPG 764 KB)

Additional file 4 Figure S4 A 99-gene metabolic plasticity signature derived from BC-K562 cells under hypoxia discriminates between BC and CP patients. a) Heatmap showing unsupervised classification by hierarchical clustering of six BC-K562 transcriptomes, showing that a 99-gene MP-signature discriminates between BC-K562 cells under hypoxia and normoxia. The gene names on the left are a random sample of the 99. The complete list is shown in supplemental Table 6, and the complete classification with expression values is shown in supplemental Table 7. b) Contingency table exploring association between clinical categories CP, AP and BC, and unsupervised classification of 113 transcriptomes from CML patients, using a 99-gene metabolic plasticity signature obtained from BC-K562 cells under hypoxia. Pie charts illustrates how the three clinical categories fit in three main “metabolic” clusters (Cluster_1, Cluster_2, and Cluster_3). Although 94% BC cases matched Cluster_2, CP cases were well represented in Cluster_1 and Cluster_3, and 85% of CP patients matched a joined single Cluster_1 + 3. c) Heatmap showing expression profile with unsupervised classification of 113 CML patients using the 99-gene metabolic plasticity signature obtained from BC-K562 cells under hypoxia. BC patients were clearly associated to the profiles of Cluster_2 while CP patients were associated to profiles of Cluster_1 and Cluster_3. (JPG 1149 KB)

Additional file 5 Figure 5. Differential expression of 25 genes in blast crisis cells within a context of metabolic reprogramming (BC vs CP) and a context of metabolic plasticity (Hypoxia vs Normoxia). Bar graph showing the sense and magnitude of differential expression of the 25 genes of the metabolic signature when used in comparison of blast crisis patients vs chronic phase patients (BC vs CP; blue bars, metabolic reprogramming), and when used in BC-K562 to compare hypoxia vs normoxia (H vs N; orange bars, metabolic plasticity). (JPG 786 KB)

Additional file 6 Figure 6. Differential expression of genes in BC-K562 cells treated with VPA and ATO. a) Differential expression of genes on BC-K562 cells exposed to 1.5 μM ATO during 48 h under normoxia. We explored a microarray assay dataset of BC-K562 cells treated with ATO during 24 h and 48 h with 1.5 μM ATO that were deposited in GEO database (GSE24946). We explored two ATO-treated samples, and two untreated samples (GSM613206, GSM613207, GSM613210, GSM613211). Enriched GO terms that could be relevant for interpreting ATO effect through DEG were “response to apoptosis”, “cellular metabolic process”, “lipid metabolism process” and “protein metabolic process”, “lysosome organelle”, “mitochondrion”, “autophagosome”, and “oxidoreductase” molecular function. Among enriched Kegg pathways we noted “metabolic pathways”, “autophagy”, “lysosome”, “carbon metabolism”, and “PPAR signalling” that may include mitochondrial biogenesis and lipid metabolism through PPARγ. GSEA analysis of these four samples showed some enriched hallmark gene lists including “ROS pathway”, “oxidative phosphorylation” and “MYC targets”. Some relevant DEG or genes on the leading edge of highly enriched gene hallmark lists were: GPX4, SOD1 (ROS), NDUFA3, MRPL11, ATP5F1D, IDH3A, NDUFB5, MDH2, COX6C, UQCRC2, NDUFV2, ETFA, FDX1, COX8A, GLS and NDUFS1 (ETC and Krebs cycle), FADS1, HMGCL, HMGCR, FASN, SLC27A5 (lipid metabolism), LAMP2, MAP1B, MAP1LC3B (lysosome activity and autophagy), HIF1A, SLC2A1/GLUT1 and MYC. The RNA expression of these restricted set of 27 genes related to mitochondria and metabolism, was sufficient to distinguish control and ATO-treated samples through hierarchical clustering, as shown in the heatmap graph. Therefore, changes in RNA expression induced by ATO treatment in BC-K562 cells, supports the notion that ATO targets mitochondria and several metabolic pathways that intersect at the mitochondria, and increases ROS under normoxia. b) Differential expression of genes on BC-K562 cells exposed to 1 mM VPA for 72 h under normoxia. We explored a microarray dataset of BC-K562 cells treated with 1 mM VPA that were deposited in GEO database (GSE19939). We explored two VPA-treated samples and two untreated samples (GSM498239, GSM498240, GSM498241, and GSM498242). Analysis of DEG showed enrichment of several biological processes (BP) GO terms related to metabolism (“cellular metabolic process”, “positive regulation of cellular biosynthetic process”, “primary metabolic process”, “positive regulation of RNA biosynthetic process”, “positive regulation of macromolecule biosynthetic process”, “positive regulation of transcription”, cellular component (CC) Go terms of intracellular locations and organelles including “mitochondrion and mitochondrial part”, and molecular function (MF) GO terms such as “transcription factor binding”, “chromatin binding”, “enzyme binding”, “histone binding”, “RNA polymerase II sequence-specific DNA binding”, “transcription factor binding”, and “nucleic acid binding”. Among enriched Kegg pathways we noted “Citrate cycle (TCA cycle)”, “Carbon metabolism”, “FoxO signalling pathway”, “PI3K-Akt signalling pathway”, and pathways including genes potentially involved in mitochondrial quality control such as “Parkinson disease”, “Pathways of neurodegeneration—multiple diseases”, and “Mitophagy– animal”. GSEA analysis of VPA-treated cells showed enrichment of several Hallmark genes related to metabolism and cell growth such “adipogenesis”, “cholesterol metabolism”, “fatty acid metabolism”, “MTORC1 signalling” and “MYC targets”. We selected 27 metabolism and mitochondria-related genes with high rank on enrichment core in GSEA or DEGs that included: HMGCR, HMGCS2, CROT, MLYCD, ALDH5A1, LDHA, PDHA1, G6PC3, HIF1A (lipid and carbohydrate metabolism), PPARGC1A, MPC1, IDH1, SLC25A17, ACOX2, GOT2, COX16, COX18, COX19 (mitochondria and metabolism), SOD2, TXNRD2, TXN2 (redox homeostasis), FOXO1, EIF4H, SP3 (transcription factors), LAMP5, GABARAPL2 and FUNDC2 (autophagy and mitophagy). The RNA expression of these restricted set of 27 genes related to mitochondria, mitochondria-related cell stress responses, and metabolisms, was sufficient to distinguish control and VPA-treated samples through hierarchical clustering as shown in the heatmap graph. Therefore, changes in RNA expression induced by VPA treatment in BC-K562 cells, supports the notion that the rescuing effects of VPA over hypoxia-induced cell death could be due to increased mitochondrial biogenesis and mitophagy (turnover and increased mitochondrial quality) and the fostering of several anabolic pathways to support growth, such as glycolysis and lipid metabolism, redox homeostasis and adaptation to hypoxia. (JPG 402 KB)

Additional file7 (XLSX 287 KB)

Additional file8 (DOCX 18 KB)

Additional file9 (DOCX 19 KB)

Additional file10 (XLSX 765 KB)

Additional file11 (XLSX 198 KB)

Additional file12 (XLSX 14 KB)

Additional file13 (XLSX 17 KB)

Additional file14 (XLSX 12 KB)

## Data Availability

The datasets generated during and/or analysed during the current study are available in the Gene Expression Omnibus (GEO) repository, [https://www.ncbi.nlm.nih.gov/geo/query/acc.cgi?acc=GSE144527; https://www.ncbi.nlm.nih.gov/geo/query/acc.cgi?acc=GSE4170], are included in supplementary information files, and are available from the corresponding author on reasonable request**.**
