# Supplementary figures and images for: Metabolic plasticity in blast crisis-chronic myeloid leukaemia cells under hypoxia reduces the cytotoxic potency of drugs targeting mitochondria
Source: Discov Oncol. 2022 Jul 8;13:60. doi: 10.1007/s12672-022-00524-y (PMC9270554; doi:10.1007/s12672-022-00524-y)

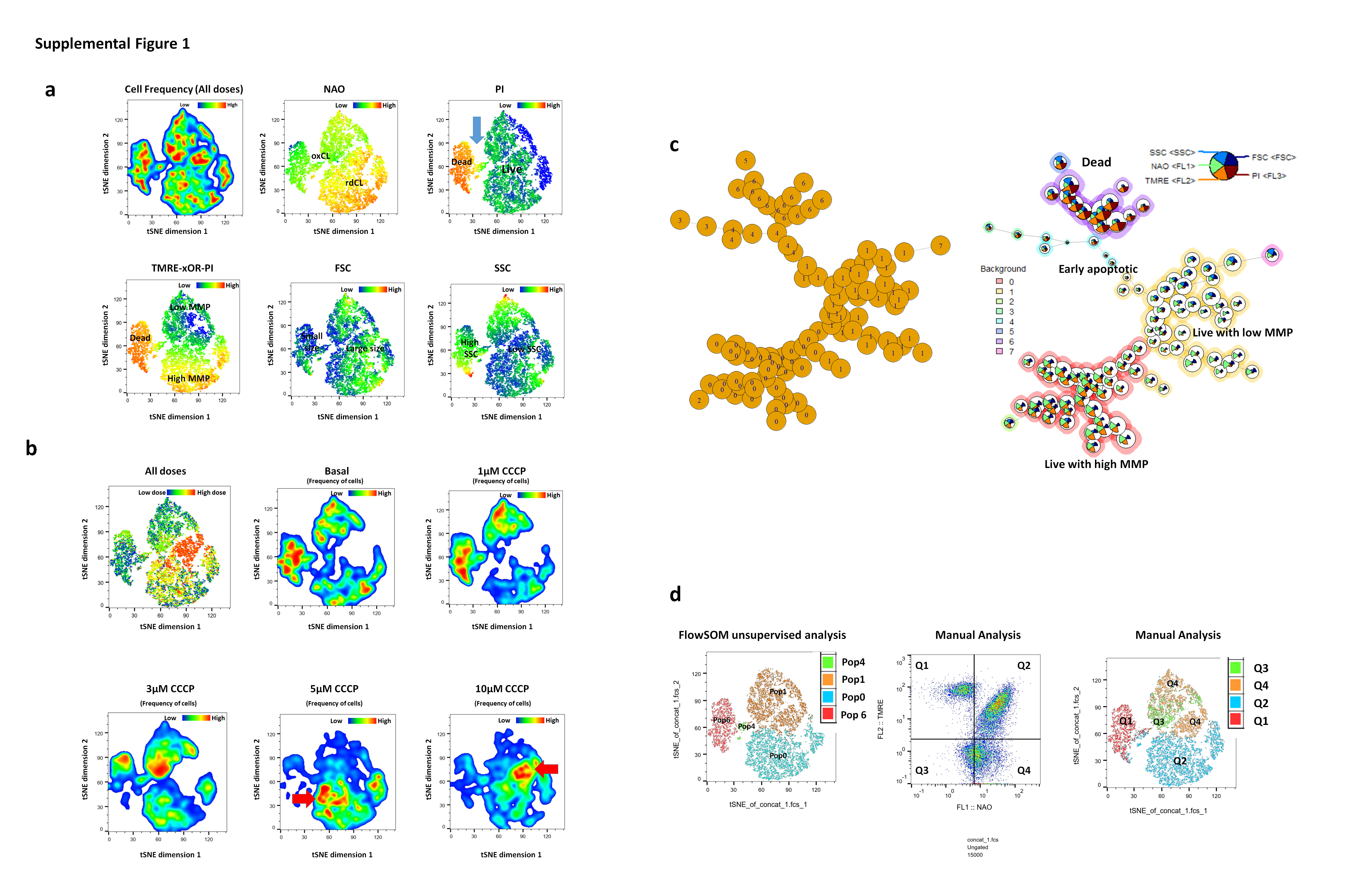

Supplement: Supplementary file 1 — Additional file 1 Fig. S1Unsupervised classification through flow cytometry self-organized maps (FlowSOM) algorithm, and visualization of the five dimensional space (FSC, SSC, NAO, TMRE-XOR-PI, and PI) through tSNE plots was in agreement with the manual quadrant analysis. a) Visualization of the five-dimensional phenotypic space (FSC, SSC, NAO, TMRE-XOR-PI, and PI) of CCCP-treated BC-K562 under hypoxia through tSNE plots. Samples corresponding to untreated cells and CCCP-treated cells with 1, 3, 5 and 10 μM CCCP were combined in a single dataset. Each point represents a single cell in the five-dimensional space. Similar cells are close in the two-dimensional t-SNE plot, while distant clusters correspond to phenotypically different groups of cells. A third parameter is represented as a heatmap colour code (NAO, TMRE-xOR-PI, PI, FSC, and SSC) to identify what population represents each cluster (annotation). Cells with high NAO (rdCL) were split in two groups with high and low MMP. Live cells and dead cells as indicated by PI content were dissimilar (distant), and a smaller transitional group of live cells was observed in between both large groups (arrow). b) Visualization of cells corresponding to untreated samples and CCCP-treated samples in the tSNE plot. The rescuing effect from hypoxia-induced death at increasing doses of CCCP is show as a transition from the “dead” cells cluster to the live cells clusters, by representing only cells from each sample at a time. At 5 μM CCCP the high MMP cluster is the larger cluster, while at 10 μM CCCP, the low MMP cluster is the largest (red arrows). In both cases the clusters corresponded to live cells with high NAO signal (rdCL) as shown in panel a). c) Unsupervised classification of the same dataset of cells shown in panels a) and b) conducted through the FlowSOM algorithm using five parameters (FSC, SSC, NAO, TMRE-XOR-PI, and PI). The algorithm identified 100 groups of very similar cells (very close in the five-dimensional s [file 12672_2022_524_MOESM1_ESM.jpg]

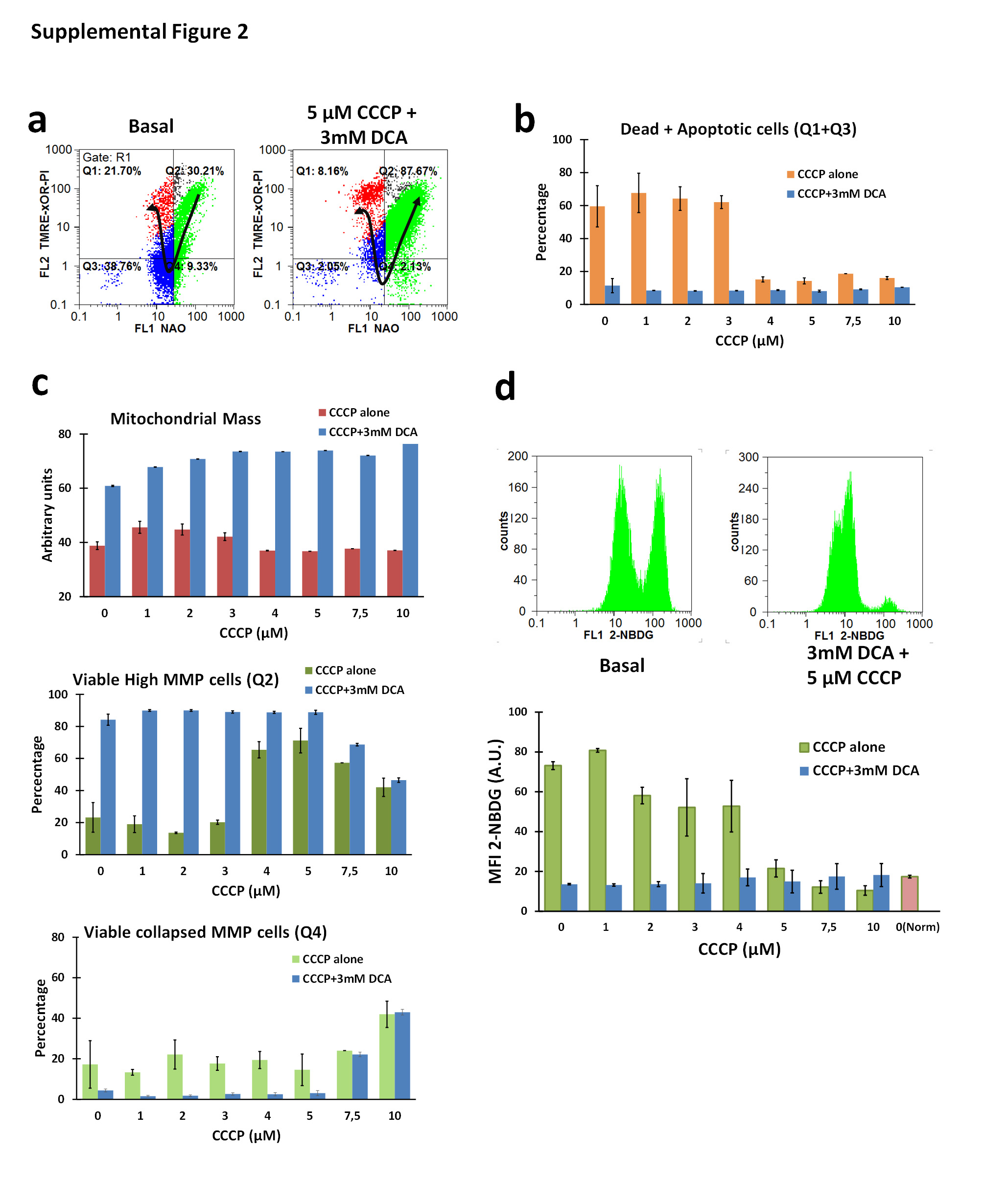

Supplement: Supplementary file 2 — Additional file 2 Fig. 2. DCA combined with CCCP rescued cells from hypoxia-induced death at 72 h in BC-K562 cells. a) Representative dot plots of 3 mM DCA + 5 μM CCCP-treated cells under hypoxia. Untreated cells show about 60% dead and apoptotic cells (red and blue dots respectively), but were rescued by combined drugs. b) Bar graph showing pro-survival effect of 3 mM DCA combined with all doses of CCCP tested (0-10 μM CCCP) under hypoxia, achieving less than10% dead + apoptotic (Q1 + Q3). c) Bar graphs showing increased MM in rescued cells with combined DCA + CCCP. About 40% of rescued cells had collapsed MMP with 10 μM CCCP + 3 mM DCA. d) Two representative histograms of cells labelled with 2-NBDG, and a bar graph showing strong decrease in glucose uptake within 3 mM DCA combined with all CCCP doses tested (0-10 μM CCCP), in all cases approaching values comparable to normoxia. MFI: mean fluorescence intensity; A.U. arbitrary units. (JPG 654 KB) [file 12672_2022_524_MOESM2_ESM.jpg]

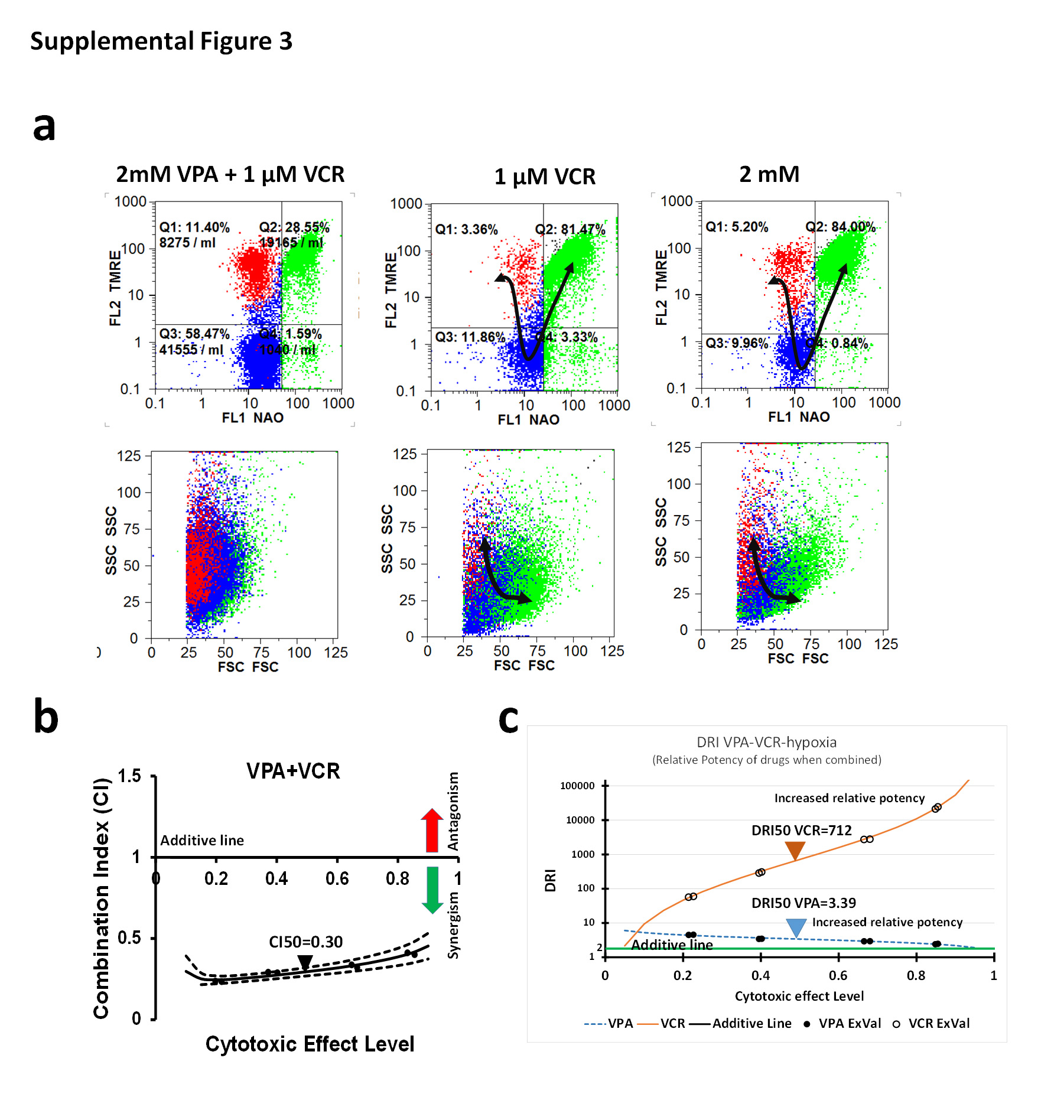

Supplement: Supplementary file 3 — Additional file 3 Fig. 3. VPA and VCR are synergic and highly cytotoxic against BC-K562 cells when combined under hypoxia. a) Representative dot plots of single and combined VPA and VCR treatment of BC-K562 cells under hypoxia. Combined treatment has no rescuing effect from hypoxia-induced death and appears highly cytotoxic. b) VPA and VCR were highly synergic with combination index (CI) well below 1.0 along the entire effect range and a value of 0.30 at 50% effect level (CI50). Assessment of interaction through CI method: For each cytotoxic level i, the combination index (CI) was calculated as: CI (i) = Dac(i) /Das(i) + Dbc(i) /Dbs(i), where Dac(i) and Dbc(i) are the doses of drugs a and b respectively required in the combination a + b to produce an effect level i. Das(i) and Dbs(i) are the doses of drug a and b respectively, required to produce an effect level i when used as single drugs. c) Drug reduction index (DRI) plot showing the increase in potency particularly of VCR with a value of 712 at 50% cytotoxic effect (DRI50). Assessment of interaction through DRI method: For each cytotoxic level i, the dose reduction index (DRI) for drugs a and b DRIa(i) and DRIb(i) were calculated as: DRIa(i) = Das(i) /Dac(i) and DRIb(i) = Dbs(i) /Dbc(i). Calculations were performed with the software Calcusyn (Biosoft, UK), which implements the above formulas [25, 31] (JPG 764 KB) [file 12672_2022_524_MOESM3_ESM.jpg]

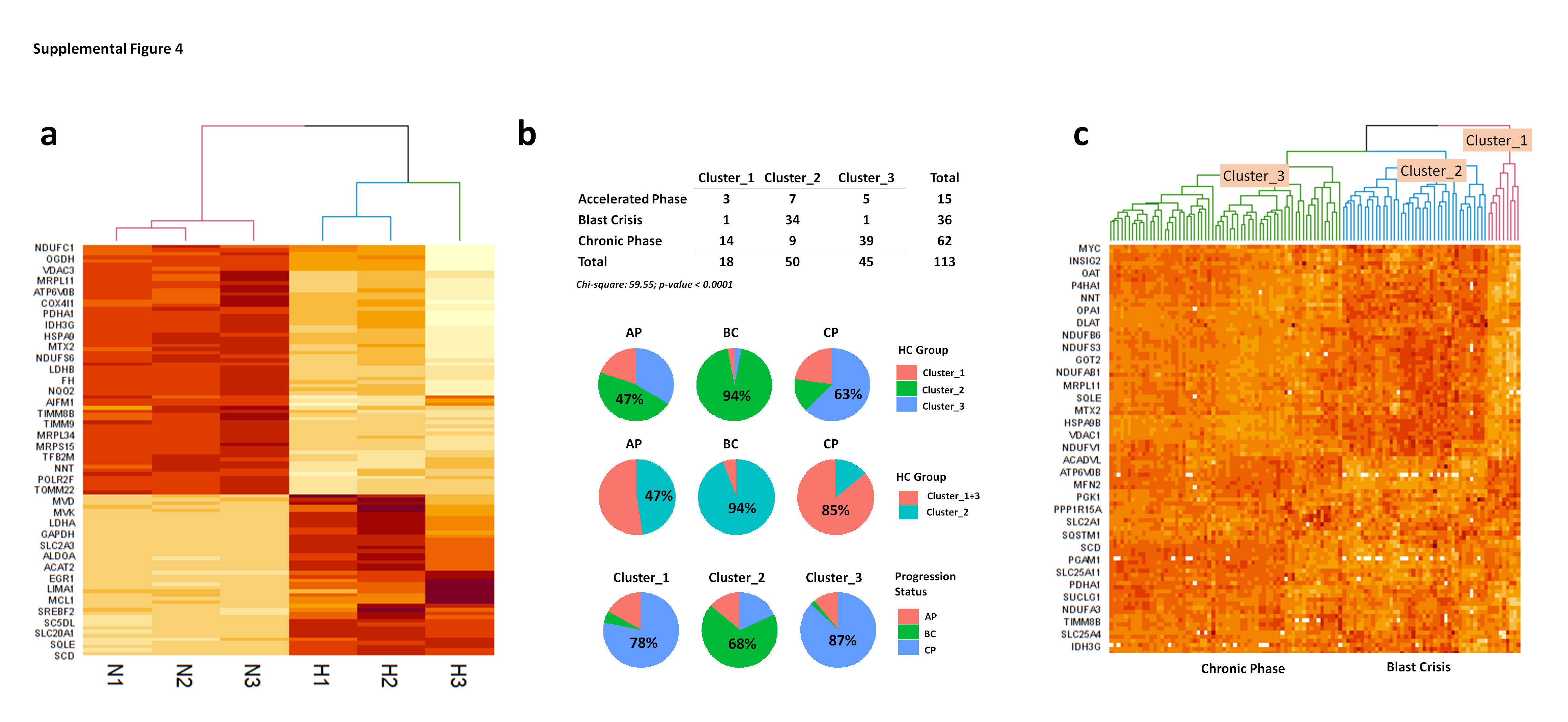

Supplement: Supplementary file 4 — Additional file 4 Figure S4 A 99-gene metabolic plasticity signature derived from BC-K562 cells under hypoxia discriminates between BC and CP patients. a) Heatmap showing unsupervised classification by hierarchical clustering of six BC-K562 transcriptomes, showing that a 99-gene MP-signature discriminates between BC-K562 cells under hypoxia and normoxia. The gene names on the left are a random sample of the 99. The complete list is shown in supplemental Table 6, and the complete classification with expression values is shown in supplemental Table 7. b) Contingency table exploring association between clinical categories CP, AP and BC, and unsupervised classification of 113 transcriptomes from CML patients, using a 99-gene metabolic plasticity signature obtained from BC-K562 cells under hypoxia. Pie charts illustrates how the three clinical categories fit in three main “metabolic” clusters (Cluster_1, Cluster_2, and Cluster_3). Although 94% BC cases matched Cluster_2, CP cases were well represented in Cluster_1 and Cluster_3, and 85% of CP patients matched a joined single Cluster_1 + 3. c) Heatmap showing expression profile with unsupervised classification of 113 CML patients using the 99-gene metabolic plasticity signature obtained from BC-K562 cells under hypoxia. BC patients were clearly associated to the profiles of Cluster_2 while CP patients were associated to profiles of Cluster_1 and Cluster_3. (JPG 1149 KB) [file 12672_2022_524_MOESM4_ESM.jpg]

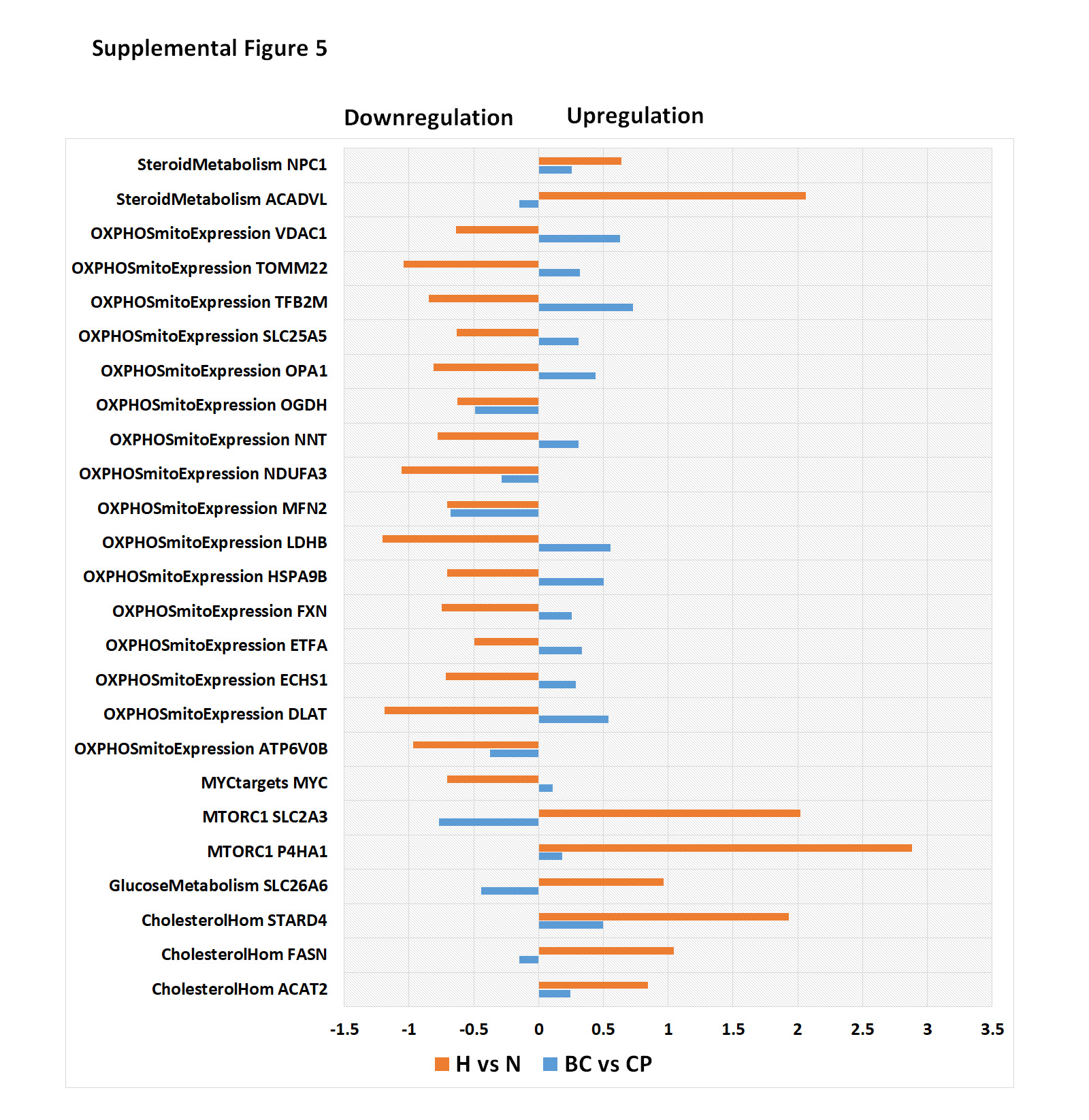

Supplement: Supplementary file 5 — Additional file 5 Figure 5. Differential expression of 25 genes in blast crisis cells within a context of metabolic reprogramming (BC vs CP) and a context of metabolic plasticity (Hypoxia vs Normoxia). Bar graph showing the sense and magnitude of differential expression of the 25 genes of the metabolic signature when used in comparison of blast crisis patients vs chronic phase patients (BC vs CP; blue bars, metabolic reprogramming), and when used in BC-K562 to compare hypoxia vs normoxia (H vs N; orange bars, metabolic plasticity). (JPG 786 KB) [file 12672_2022_524_MOESM5_ESM.jpg]

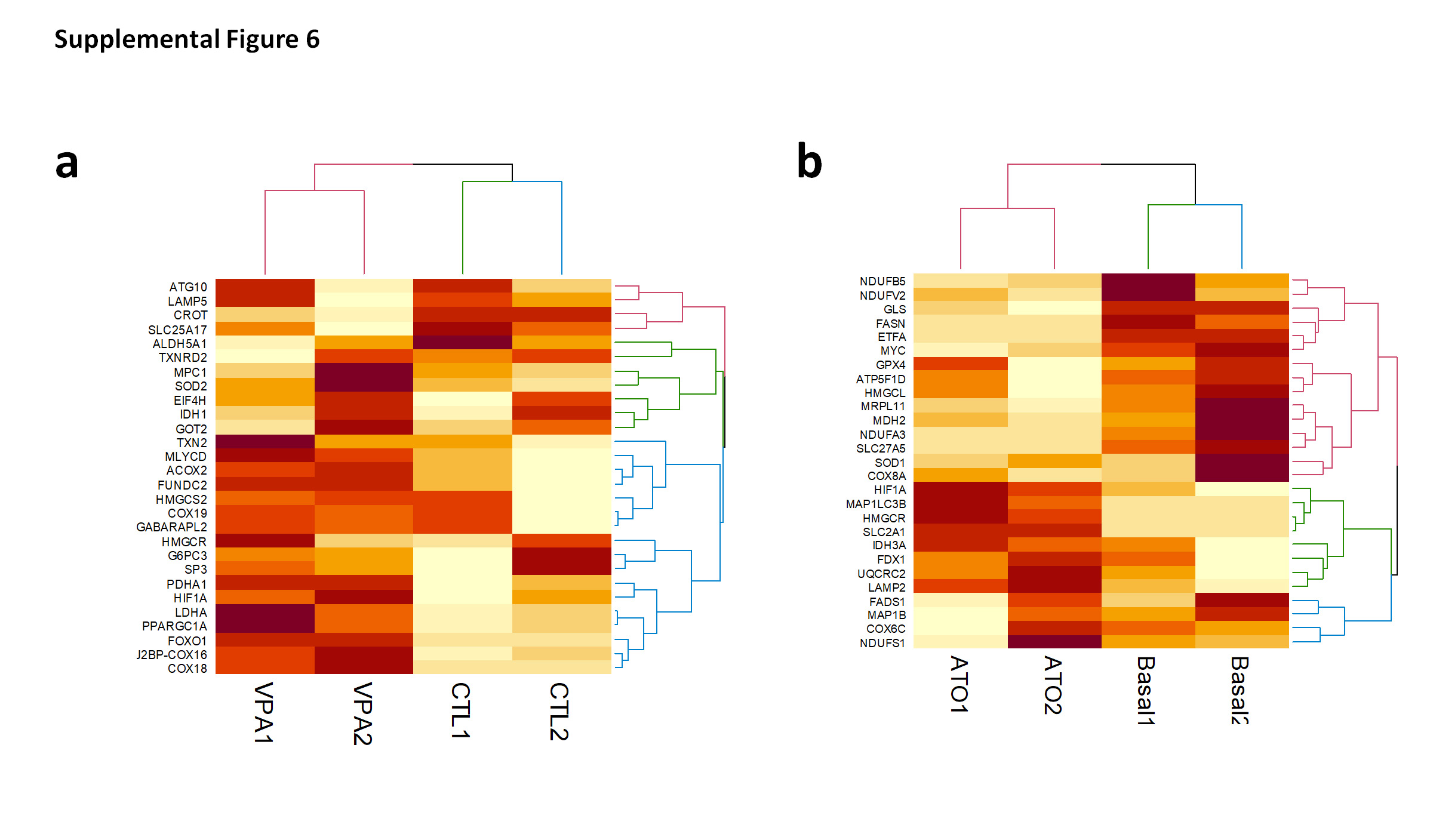

Supplement: Supplementary file 6 — Additional file 6 Figure 6. Differential expression of genes in BC-K562 cells treated with VPA and ATO. a) Differential expression of genes on BC-K562 cells exposed to 1.5 μM ATO during 48 h under normoxia. We explored a microarray assay dataset of BC-K562 cells treated with ATO during 24 h and 48 h with 1.5 μM ATO that were deposited in GEO database (GSE24946). We explored two ATO-treated samples, and two untreated samples (GSM613206, GSM613207, GSM613210, GSM613211). Enriched GO terms that could be relevant for interpreting ATO effect through DEG were “response to apoptosis”, “cellular metabolic process”, “lipid metabolism process” and “protein metabolic process”, “lysosome organelle”, “mitochondrion”, “autophagosome”, and “oxidoreductase” molecular function. Among enriched Kegg pathways we noted “metabolic pathways”, “autophagy”, “lysosome”, “carbon metabolism”, and “PPAR signalling” that may include mitochondrial biogenesis and lipid metabolism through PPARγ. GSEA analysis of these four samples showed some enriched hallmark gene lists including “ROS pathway”, “oxidative phosphorylation” and “MYC targets”. Some relevant DEG or genes on the leading edge of highly enriched gene hallmark lists were: GPX4, SOD1 (ROS), NDUFA3, MRPL11, ATP5F1D, IDH3A, NDUFB5, MDH2, COX6C, UQCRC2, NDUFV2, ETFA, FDX1, COX8A, GLS and NDUFS1 (ETC and Krebs cycle), FADS1, HMGCL, HMGCR, FASN, SLC27A5 (lipid metabolism), LAMP2, MAP1B, MAP1LC3B (lysosome activity and autophagy), HIF1A, SLC2A1/GLUT1 and MYC. The RNA expression of these restricted set of 27 genes related to mitochondria and metabolism, was sufficient to distinguish control and ATO-treated samples through hierarchical clustering, as shown in the heatmap graph. Therefore, changes in RNA expression induced by ATO treatment in BC-K562 cells, supports the notion that ATO targets mitochondria and several metabolic pathways that intersect at the mitochondria, and increases ROS under normoxia. b) Differential expression of genes on BC-K [file 12672_2022_524_MOESM6_ESM.jpg]
