# Supplementary material for: Metabolic plasticity in blast crisis-chronic myeloid leukaemia cells under hypoxia reduces the cytotoxic potency of drugs targeting mitochondria
Source: Discov Oncol. 2022 Jul 8;13:60. doi: 10.1007/s12672-022-00524-y (PMC9270554; doi:10.1007/s12672-022-00524-y)
Supplement: Supplementary file 8 — Additional file8 (DOCX 18 KB) [file 12672_2022_524_MOESM8_ESM.docx]

**Supplemental Table 2**

**Gene Ontology (GO) terms with differentially expressed genes in BC-K562 cells under hypoxia**

|  | **Term** | **Ont** | **N** | **Down** | **Up** | **P.Down** | **P.Up** |
| --- | --- | --- | --- | --- | --- | --- | --- |
| GO:0005759 | **mitochondrial matrix** | CC | 248 | 104 | 11 | 1.22E-20 | 1.00E+00 |
| GO:0005743 | **mitochondrial inner membrane** | CC | 247 | 99 | 10 | 5.57E-18 | 1.00E+00 |
| GO:0098798 | **mitochondrial protein complex** | CC | 174 | 78 | 2 | 1.25E-17 | 1.00E+00 |
| GO:0140053 | **mitochondrial gene expression** | BP | 115 | 59 | 4 | 6.00E-17 | 9.99E-01 |
| GO:0019866 | **organelle inner membrane** | CC | 263 | 101 | 11 | 8.61E-17 | 1.00E+00 |
| GO:0005761 | **mitochondrial ribosome** | CC | 73 | 44 | 3 | 2.23E-16 | 9.92E-01 |
| GO:0000313 | **organellar ribosome** | CC | 73 | 44 | 3 | 2.23E-16 | 9.92E-01 |
| GO:0005739 | **mitochondrion** | CC | 652 | 191 | 53 | 3.86E-16 | 9.98E-01 |
| GO:0032543 | **mitochondrial translation** | BP | 105 | 54 | 4 | 1.18E-15 | 9.98E-01 |
| GO:0022613 | **ribonucleoprotein complex biogenesis** | BP | 272 | 101 | 8 | 1.28E-15 | 1.00E+00 |
| GO:0070125 | **mitochondrial translational elongation** | BP | 76 | 44 | 2 | 1.85E-15 | 9.99E-01 |
| GO:0006415 | **translational termination** | BP | 85 | 47 | 2 | 2.30E-15 | 1.00E+00 |
| GO:0005740 | **mitochondrial envelope** | CC | 350 | 118 | 22 | 1.57E-14 | 1.00E+00 |
| GO:0042254 | **ribosome biogenesis** | BP | 181 | 74 | 6 | 3.93E-14 | 1.00E+00 |
| GO:0070126 | **mitochondrial translational termination** | BP | 76 | 42 | 2 | 7.72E-14 | 9.99E-01 |
| GO:0031966 | **mitochondrial membrane** | CC | 328 | 111 | 20 | 8.21E-14 | 1.00E+00 |
| GO:0003723 | **RNA binding** | MF | 894 | 232 | 51 | 5.49E-13 | 1.00E+00 |
| GO:0050896 | **response to stimulus** | BP | 1892 | 317 | 284 | 9.14E-01 | 6.61E-13 |
| GO:0070013 | **intracellular organelle lumen** | CC | 1943 | 428 | 172 | 8.35E-13 | 1.00E+00 |
| GO:0031974 | **membrane-enclosed lumen** | CC | 1943 | 428 | 172 | 8.35E-13 | 1.00E+00 |
| GO:0043233 | **organelle lumen** | CC | 1943 | 428 | 172 | 8.35E-13 | 1.00E+00 |
| GO:0034641 | **cellular nitrogen compound metabolic process** | BP | 1944 | 427 | 165 | 1.68E-12 | 1.00E+00 |
| GO:1990904 | **ribonucleoprotein complex** | CC | 459 | 138 | 15 | 1.92E-12 | 1.00E+00 |
| GO:0034660 | **ncRNA metabolic process** | BP | 221 | 80 | 3 | 7.84E-12 | 1.00E+00 |
| GO:0051716 | **cellular response to stimulus** | BP | 1558 | 256 | 242 | 9.44E-01 | 9.35E-12 |
| GO:0070887 | **cellular response to chemical stimulus** | BP | 776 | 129 | 143 | 8.01E-01 | 1.66E-11 |
| GO:0031975 | **envelope** | CC | 517 | 148 | 36 | 1.83E-11 | 1.00E+00 |
| GO:0031967 | **organelle envelope** | CC | 517 | 148 | 36 | 1.83E-11 | 1.00E+00 |
| GO:0071944 | **cell periphery** | CC | 769 | 91 | 140 | 1.00E+00 | 7.94E-11 |
| GO:0006414 | **translational elongation** | BP | 96 | 44 | 3 | 8.80E-11 | 9.99E-01 |
| GO:0050794 | **regulation of cellular process** | BP | 2281 | 338 | 319 | 1.00E+00 | 1.04E-10 |
| GO:0050789 | **regulation of biological process** | BP | 2442 | 363 | 335 | 1.00E+00 | 1.84E-10 |
| GO:0042221 | **response to chemical** | BP | 999 | 166 | 169 | 8.39E-01 | 1.93E-10 |
| GO:0065007 | **biological regulation** | BP | 2582 | 391 | 349 | 1.00E+00 | 2.28E-10 |
| GO:0005762 | **mitochondrial large ribosomal subunit** | CC | 46 | 27 | 0 | 3.88E-10 | 1.00E+00 |
|  | | | | | | | |

(UP) Genes upregulated in hypoxia compared to normoxia

(Down) Genes downregulated in hypoxia compared to normoxia

(N) Number of genes in the GO term

(0nt) CC: cellular component; BP: biological process; MF: molecular function

(P.Up) p-value for enrichment of GO term in up-regulated genes.

(P.Down) p-value for enrichment of GO term in down-regulated genes.

This output was obtained with GOANA function of limma R package that performs an enrichment analyses for Gene Ontology terms. The entire fitted linear model (fit object) as obtained with lmFit and eBayes functions of limma R package, containing all genes probed, was used as input for GOANA function, using the default FDR cut-off parameter value of 0.05 [34].
