# Supplementary material for: Metabolic plasticity in blast crisis-chronic myeloid leukaemia cells under hypoxia reduces the cytotoxic potency of drugs targeting mitochondria
Source: Discov Oncol. 2022 Jul 8;13:60. doi: 10.1007/s12672-022-00524-y (PMC9270554; doi:10.1007/s12672-022-00524-y)
Supplement: Supplementary file 9 — Additional file9 (DOCX 19 KB) [file 12672_2022_524_MOESM9_ESM.docx]

**Supplemental Table 3**

**Kegg pathways with differentially expressed genes** **in BC-K562 cells under hypoxia**

|  | **Pathway** | **N** | **Down** | **Up** | **P.Down** | **P.Up** |
| --- | --- | --- | --- | --- | --- | --- |
| path:hsa00010 | **glycolysis / gluconeogenesis** | 29 | 4 | 13 | 7.78E-01 | 4.85E-06 |
| path:hsa04510 | **focal adhesion** | 43 | 0 | 16 | 1.00E+00 | 7.46E-06 |
| path:hsa03008 | **ribosome biogenesis in eukaryotes** | 48 | 21 | 0 | 2.06E-05 | 1.00E+00 |
| path:hsa03030 | **DNA replication** | 30 | 15 | 1 | 4.79E-05 | 9.72E-01 |
| path:hsa00100 | **steroid biosynthesis** | 11 | 1 | 7 | 8.82E-01 | 4.80E-05 |
| path:hsa04670 | **leukocyte transendothelial migration** | 26 | 0 | 11 | 1.00E+00 | 5.17E-05 |
| path:hsa00230 | **purine metabolism** | 39 | 17 | 3 | 1.35E-04 | 8.31E-01 |
| path:hsa04810 | **regulation of actin cytoskeleton** | 57 | 4 | 16 | 9.94E-01 | 3.63E-04 |
| path:hsa00983 | **drug metabolism - other enzymes** | 19 | 10 | 2 | 5.42E-04 | 6.48E-01 |
| path:hsa00051 | **fructose and mannose metabolism** | 15 | 1 | 7 | 9.46E-01 | 6.24E-04 |
| path:hsa00900 | **terpenoid backbone biosynthesis** | 15 | 3 | 7 | 5.08E-01 | 6.24E-04 |
| path:hsa01230 | **biosynthesis of amino acids** | 39 | 10 | 12 | 1.35E-01 | 8.03E-04 |
| path:hsa04520 | **adherens junction** | 25 | 0 | 9 | 1.00E+00 | 1.04E-03 |
| path:hsa04066 | **HIF-1 signalling pathway** | 36 | 6 | 11 | 6.29E-01 | 1.41E-03 |
| path:hsa01100 | **metabolic pathways** | 458 | 104 | 64 | 1.91E-03 | 3.24E-02 |
| path:hsa03010 | **ribosome** | 118 | 33 | 4 | 3.18E-03 | 1.00E+00 |
| path:hsa00240 | **pyrimidine metabolism** | 20 | 9 | 1 | 4.11E-03 | 9.09E-01 |
| path:hsa05016 | **huntington disease** | 157 | 41 | 5 | 4.29E-03 | 1.00E+00 |
| path:hsa01200 | **carbon metabolism** | 59 | 18 | 14 | 1.04E-02 | 4.74E-03 |
| path:hsa05340 | **primary immunodeficiency** | 4 | 0 | 3 | 1.00E+00 | 5.18E-03 |
| path:hsa04010 | **MAPK signalling pathway** | 48 | 3 | 12 | 9.94E-01 | 5.57E-03 |
| path:hsa04922 | **glucagon signalling pathway** | 26 | 1 | 8 | 9.94E-01 | 6.00E-03 |
| path:hsa05135 | **Yersinia infection** | 43 | 5 | 11 | 8.98E-01 | 6.51E-03 |
| path:hsa03430 | **mismatch repair** | 18 | 8 | 1 | 7.43E-03 | 8.84E-01 |
| path:hsa04640 | **hematopoietic cell lineage** | 8 | 1 | 4 | 7.88E-01 | 7.64E-03 |
| path:hsa04213 | **longevity regulating pathway multiple species** | 15 | 7 | 0 | 8.91E-03 | 1.00E+00 |
| path:hsa04140 | **autophagy - animal** | 51 | 8 | 12 | 6.99E-01 | 9.29E-03 |
| path:hsa03040 | **spliceosome** | 99 | 27 | 3 | 1.04E-02 | 9.99E-01 |
| path:hsa00982 | **drug metabolism cytochrome P450** | 6 | 4 | 1 | 1.06E-02 | 5.11E-01 |
| path:hsa04152 | **AMPK signalling pathway** | 34 | 7 | 9 | 3.92E-01 | 1.06E-02 |
| path:hsa04216 | **ferroptosis** | 18 | 3 | 6 | 6.39E-01 | 1.11E-02 |
| path:hsa04210 | **apoptosis** | 48 | 2 | 11 | 9.99E-01 | 1.53E-02 |
| path:hsa03410 | **base excision repair** | 24 | 9 | 1 | 1.65E-02 | 9.43E-01 |
| path:hsa03020 | **RNA polymerase** | 24 | 9 | 0 | 1.65E-02 | 1.00E+00 |
| path:hsa04611 | **platelet activation** | 25 | 2 | 7 | 9.51E-01 | 1.72E-02 |

(UP) Genes upregulated in hypoxia compared to normoxia

(Down) Genes downregulated in hypoxia compared to normoxia

(N) Number of genes in Kegg pathway

(P.Up) p-value for enrichment of GO term in up-regulated genes.

(P.Down) p-value for enrichment of GO term in down-regulated genes.

This output was obtained with KEGGA function of limma R package that performs an enrichment analyses for Kegg pathways terms. The entire fitted linear model (fit object) as obtained with lmFit and eBayes functions of limma R package, containing all genes probed, was used as input for KEGGA function, using the default FDR cut-off parameter value of 0.05 [34].
